# Stereospecific Assembly of Triply Chiral Pseudopeptidic Cages Through Dynamic Dual Chirality Transfer

**DOI:** 10.1002/anie.202525964

**Published:** 2026-01-28

**Authors:** Tushar D. Bhosale, Eudald Usan, Delia Miguel, Jordi Solà, Angel Orte, Ciril Jimeno, Ignacio Alfonso

**Affiliations:** ^1^ Department of Biological Chemistry Institute For Advanced Chemistry of Catalonia IQAC‐CSIC Barcelona Spain; ^2^ Nanoscopy‐UGR Laboratory Departamento De Fisicoquímica Facultad De Farmacia Unidad de Excelencia de Química University of Granada Granada Spain

**Keywords:** chirality, constitutional dynamic chemistry, metal coordination, molecular cages, pseudopeptides

## Abstract

A stereospecific transmission of chiral information within tripodal pseudopeptidic cages has been observed from dynamic constitutional co‐operation of reversible orthogonal processes: imine bond and metal coordination. The chiral centers from the phenylalanine amino acid moieties induce a specific chirality into the formed octahedral Fe(II) complex and into the helical twist of the macrobicycle, as confirmed by NMR, circular dichroism, X‐ray diffraction of single crystals, and theoretical calculations. Instead, when a configurationally stable Ru(II) complex is employed, the dynamic imine exchange efficiently selects the corresponding matched‐combination of the same chiral elements, leading after reduction to a water‐soluble homochiral stable macrobicyclic pseudopeptide Ru(II) complex with appealing chemical and photophysical properties. Overall, our study demonstrates how subtle structural factors from a chiral effector in a reversible dynamic system rule the generation and selection of subsequent chiral elements with high fidelity and complete stereocontrol.

## Graphical abstract

The spontaneous assembly of triply chiral (L, Δ, *P* or D, Λ, *M*) cages has been achieved from a simple tripodal chiral effector based on phenylalanine tris(amidoamine), non‐chiral bipyridine dialdehyde, and a metal ion. Orthogonal imine bond and iron (II) coordination generate a single combination of chiral elements, while configurationally stable ruthenium (II) allows their stereospecific selection, reflecting a highly efficient dynamic chiral information transfer.

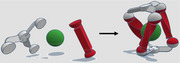

Chirality, a purely geometrical property of objects, is indeed one of the most appealing features of molecules, meaning a key structural factor in synthesis [1], catalysis [2], materials science [3, 4], and biology [5]. In living organisms, homochirality is expressed as the main ubiquitous abundance of a single enantiomer for essential chiral biomolecules (i.e., L‐amino acids and D‐carbohydrates) [6]. The perpetuation of homochirality requires a delicate transmission of chiral information during all the biological processes, such as protein expression from genetic material, DNA replication, or enzymatic catalysis [7]. Moreover, a well‐defined higher structural order sources from a specifically programmed folding where information is transferred from the central chirality of building blocks to the secondary structure of biopolymers, as observed in α‐helical peptides or different forms of nucleic acids [8]. Chemists have been fascinated by the homochirality of the natural world from the fundamental point of view, as well as for potential biomedical and biomaterials applications. In this regard, proposing mechanisms for the suitable transmission of chiral information in simple abiotic models is crucial for understanding how homochirality has evolved and succeeded [9]. In the last decades, constitutional dynamic chemistry (CDC) has emerged as a suitable approach to interrogate chemical systems about fundamental aspects like the spontaneous formation of complex molecular and supramolecular entities [10, 11]. Thus, the design of dynamic mixtures of species formed from simple building blocks (BBs) allows us to study how these living mixtures respond to external stimuli [12] or develop functions according to inner properties [13, 14, 15, 16]. During the dynamic selection process through proofreading and error correction, the right chemical information is expressed and transmitted between BBs and from them to the newly formed species [14]. We envisioned CDC to unravel new mechanisms for transmission of chiral information [17, 18], since the combination of building blocks connected by dynamic covalent and non‐covalent interactions allows the overall system to express subtle structural factors [19], like different signs and levels of chirality [20, 21]. In this work, we employed simple synthetic chiral and achiral building blocks that, after reversible interactions, can efficiently transfer chiral information, expressing a hierarchically superior ordering in a specific fashion [22]. To this aim, we decided to combine two well‐established dynamic bonds: the imine bond and transition metal coordination, but without interconnection between them to fully control their exchanging behavior. As the structural framework, we focused on pseudopeptidic tripodal cages [23], as stable structures where both chirality and binding motifs can be conveniently implemented [24, 25]. Moreover, chiral molecular cages [26] and related systems have demonstrated appealing applications in challenging stereoselective synthesis [27], solar energy conversion [28], and information transfer [29].

In a first approach, as BBs, we used the chiral effector L‐**1** (Figure 1A), made of tris(2‐aminoethyl)amine (Tren) and L‐Phe, [30] and the metal‐coordinating [2,2′‐bipyridine]‐5,5′‐dicarbaldehyde **2** [31]. In solution, these molecules can undergo both imine formation and exchange reactions, as well as orthogonal metal coordination (i.e., without direct imine bond–metal participation). As the metal center, we selected Fe(II), since the bipy‐Fe(II) binding also exchanges fast at room temperature, allowing the cooperative action and error correction of both dynamic processes. The ^1^H NMR spectrum of the equilibrated mixture showed a simplified set of sharp signals (Figure 1B), suggesting the quantitative formation of a single homochiral *D_3_*‐symmetric macrobicycle, L‐**3**Fe. The downfield chemical shifts of the bipy moiety signals and the color change of the sample indicated an efficient Fe(II) coordination that could be further confirmed by MALDI‐TOF mass spectrometry (see Supporting Information). Moreover, the strong anisochrony of all the diastereotopic methylenes (see T1/T1’, T2/T2’, and β/β’ signals in Figure 1B) of the molecule suggested a well‐defined chiral stable structure in solution. For instance, diastereotopic splitting of the methylene close to amide NH (labelled as T2/T2’) is as large as 1.25 ppm, a remarkable value for protons attached to the same carbon. Circular Dichroism spectrum of L‐**3**Fe (CD, blue solid line in Figure 1C) shows a very intense negative split‐Cotton effect with a negative minimum at 339 nm and a positive maximum at 314 nm, and an additional less intense positive Cotton effect centered at ca. 525 nm, assigned to ligand and metal‐to‐ligand transitions, respectively. These observations suggest the formation of a helical cage with a well‐defined Δ chiral configuration of the Fe(bipy)_3_ moiety [32]. The same reaction performed with the enantiomeric tripodal pseudopeptide, D‐**1**, led to the corresponding enantiomer D‐**3**Fe with a specular CD spectrum (orange in Figure 1C), meaning in this case a Λ metal‐complex configuration. Thus, there is an efficient chirality transfer from the configurations of the chiral amino acids of L‐**1** or D‐**1** that *stereospecifically induces* a Δ or Λ configuration in the metal complex, respectively. Control experiments performed in the absence of Fe(II) led to the formation of the corresponding hexaimine macrobicycle but with a lower diasterotopicity of their methylenes and less intense CD spectrum with no signs of bi‐signed bands, meaning a much less efficient chiral ordering (Figure 1C and Supporting Information).

**FIGURE 1:**
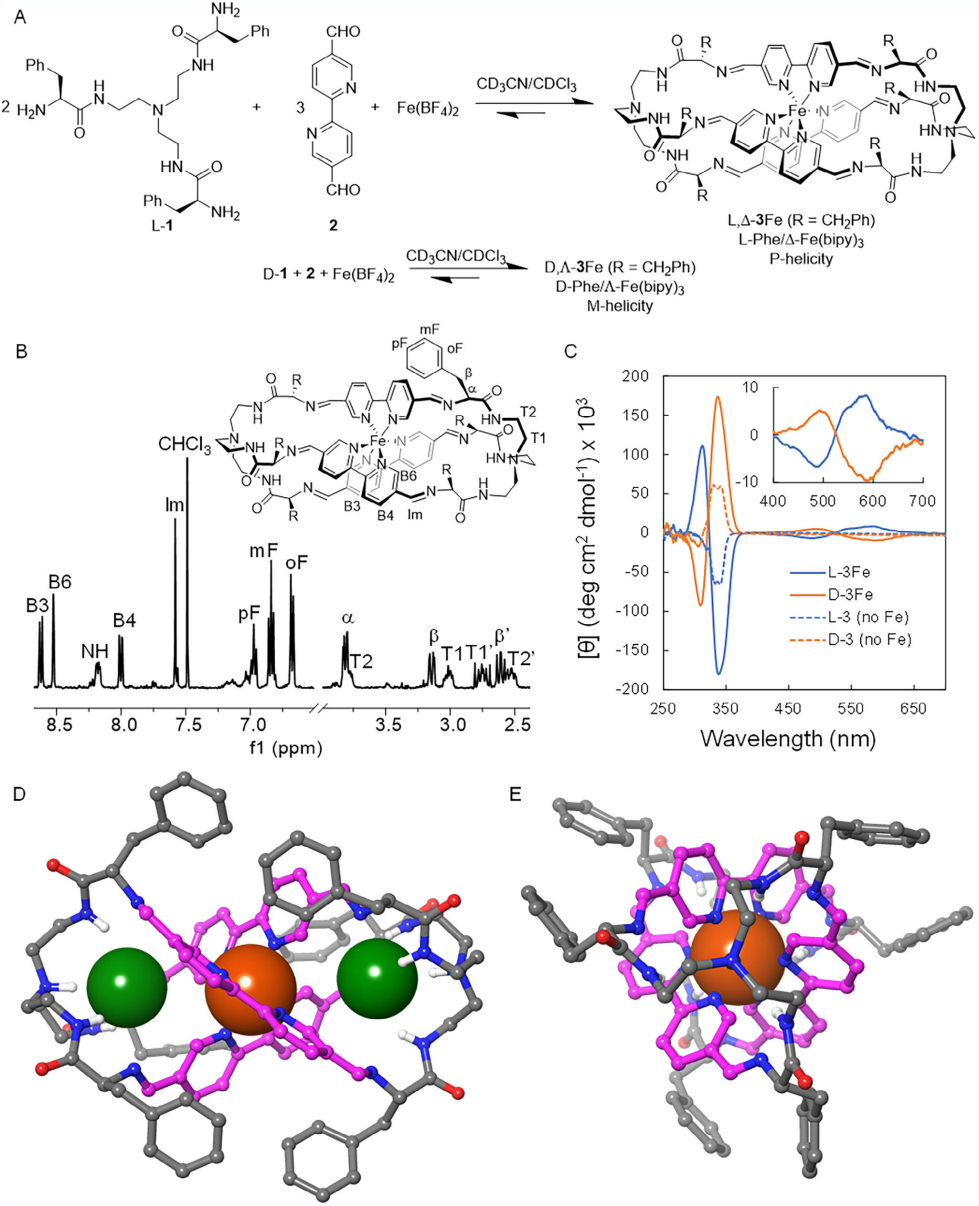
(A) Stereospecific dynamic assembly of helical Fe(II) cage with a chiral induction from the amino acid Cα centers to the metal center and helical sense. (B) ^1^H NMR spectrum of the crude reaction after equilibration depicting assignment of signals for the corresponding moieties (arbitrary labelling). (C) Circular dichroism spectra for helical L‐**3**Fe (blue solid line) and D‐**3**Fe (orange solid line), as well as the corresponding cages without Fe(II) metal (dashed traces). Inset shows a zoom of the bi‐signed signal at lower energy. (D,E) Two views of L‐**3**Fe (BF_4_)_2_·2HCl salt obtained by single crystal X‐ray diffraction. Non‐polar hydrogen atoms, BF_4_^−^ counterions, and solvent molecules found in the crystal were omitted for clarity. Iron and chloride ions are represented as space‐filling, and C‐atoms for bipyridine bis‐imines are shown in magenta. In (E), chloride has been occulted for simplicity.

After several attempts, we were able to obtain single crystals of L‐**3**Fe as the corresponding bis(tetrafluoroborate) salt suitable for X‐ray diffraction (XRD) analysis (Figure 1D,E and Supporting Information) [33]. The first striking observation was the presence of two additional chloride anions (green spheres in Figure 1D) inside the two defined cavities of the formed cryptand. It was found that adventitious HCl is sequestered by the cage, protonating the two tertiary amine groups and facilitating the encapsulation of chloride through a combination of Coulombic and hydrogen bond stabilization with the protonated amine and the tripodal amide [34, 35, 36]. Considering these observations, we repeated the assembling of the cage in the presence of two equivalents of tetrabutylammonium chloride, showing no differences with respect to the reaction in the absence of added chloride. Regarding the iron coordination, a perfect octahedral complex with Δ configuration was obtained (Figure 1D), confirming the assignment done by CD and the L/Δ‐matching combination of chiral elements. Moreover, the molecule shows P‐helicity [37] through the winding of the three cage bars around the *C_3_* symmetry axis connecting [N^+^─H···Cl···Fe···Cl···H─N^+^] atoms (Figure 1E), which represents a third level of stereocontrol that correlates with the well‐defined chiral structure observed in solution. The amino acid side chains are folded over the grooves defined by the bipy moieties, possibly establishing edge‐to‐face aromatic‐aromatic interactions. The proximity between aromatic rings would explain the upfield shift observed for the phenyl rings of the side chains in the ^1^H NMR spectrum of L‐**3**Fe (Figure 1B). Thus, signals from ortho (oF), meta (mF), and para (pF) protons of the phenyl side chains are shielded by 0.41, 0.33, and 0.13 ppm, respectively, with respect to the precursor L‐**1** ^1^H NMR spectrum in the same solvent mixture. Additional proof for the side chain folding in solution was obtained by clear cross peaks between phenyl and bipy proton signals in the 2D ROESY spectrum of L‐**3**Fe (Supporting Information). Overall, both in solid and in solution states, the structure is a highly compact helical Fe(II) cage that confirms how the chiral information contained in the chiral centers of the amino acids of the pseudopeptidic tris(amidoamine) induces the formation of a single chirality of the metal center and a higher order helicity of the whole molecule, fulfilling a stereospecific information transfer [17].

To test the robustness of the chiral induction process, we designed competition experiments using a *pseudo*‐racemic mixture of the chiral inducer. Accordingly, we mixed equimolar amounts of D‐**1** with the corresponding tris(amidoamine) L‐**1** ^F^ (Figure 2), prepared from Tren and L‐*ortho*‐fluorophenylalanine. Under the optimized reaction conditions with **2** in the presence of Fe(II) and after equilibration, we observed by MALDI‐TOF mass spectrometry (Figure 2) the formation of two homochiral iron cages either without fluorine or containing six F atoms, implying the complete chiral self‐sorting of the process. Control experiments carried out with a mixture of L‐**1** and L‐**1** ^F^ led to the three possible combinations (i.e., with zero, three, and six F atoms), confirming the chirality‐driven self‐sorting [38, 39, 40]. The corresponding experiments using the *para*‐F‐substituted amino acid also rendered the same results, ruling out any effect of fluorine substitution in the selection (see Supporting Information). These observations demonstrate that the dynamic self‐selection process efficiently transfers the chiral information not only from the chiral inducer to the metal complex, but also between chiral inducers, most likely through the dynamic metal complex with the *matched* stereochemistry.

**FIGURE 2:**
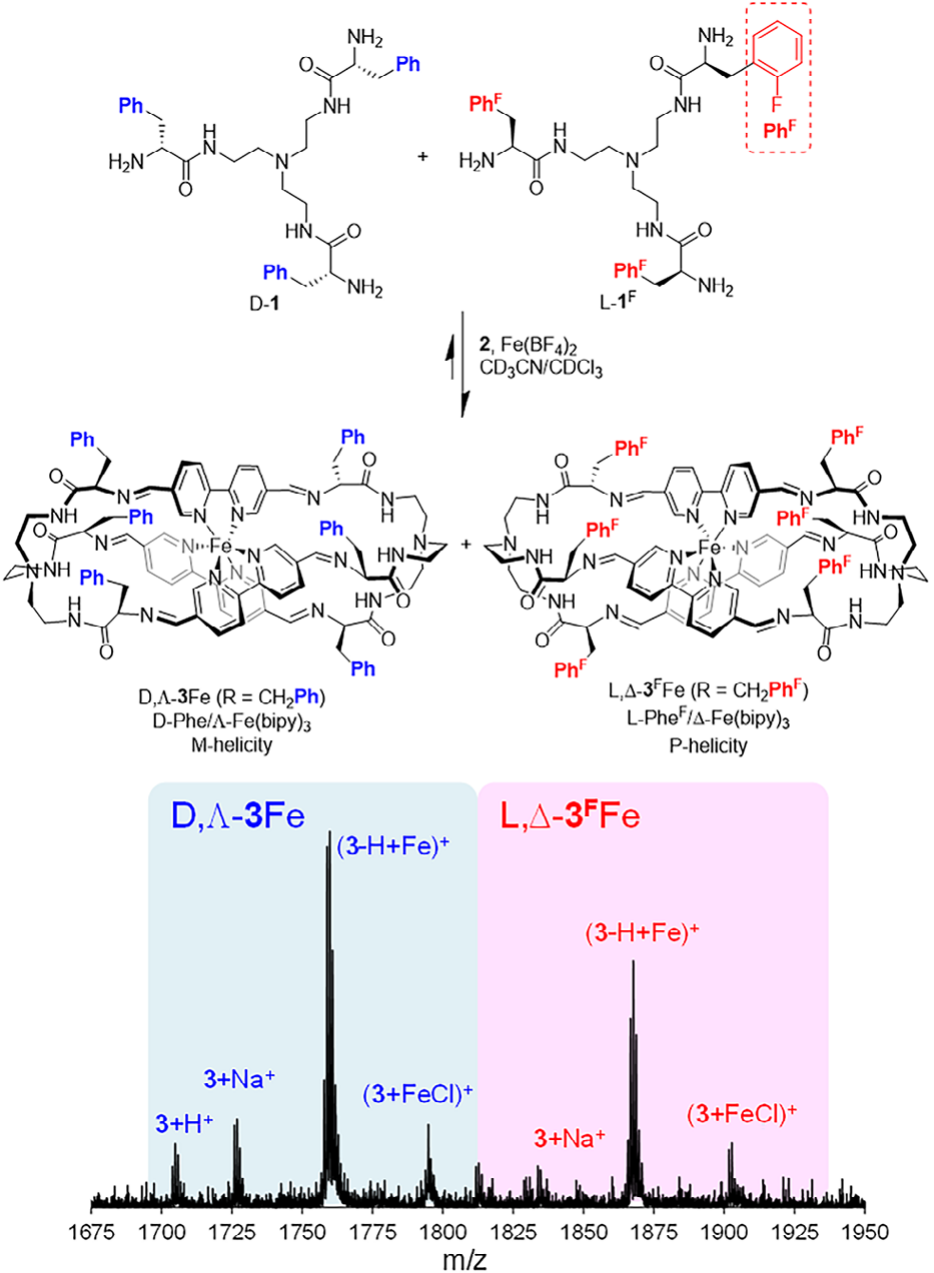
Chiral self‐sorting experiment with pseudo‐enantiomers of pseudopeptidic triamines.

In order to better understand the source of the observed selectivity, we performed DFT calculations in implicit acetonitrile (the main solvent of the reaction). First, we optimized the corresponding Fe(II)‐cage bearing L‐Phe and Δ‐Fe(II) configurations (Figure 3A). The energy minimum of the dicationic Fe(II) complex is very similar to the structure experimentally found in the solid state, suggesting that the two HCl molecules are not essential for the stable assembly in solution. Each Phe aromatic side chain folds onto the closest helix groove, establishing edge‐to‐face aromatic interactions (blue dashed lines in Figure 3A) with the two rings of the nearby bipy. This arrangement sets the *ortho* and *meta* protons of Phe residues pointing to the two aromatic rings of bipy, explaining the upfield shifts and ROEs observed in NMR spectra. Then, we built the *mismatched* combinations of diastereomers by consecutively inverting the configuration of the tripodal pseudopeptidic moieties to D‐Phe, but retaining the Δ configuration of the metal complex. DFT calculations rendered lesser stable minima by 27.0 and 43.8 kcal/mol for the D‐Phe//Δ‐Fe//L‐Phe and D‐Phe//Δ‐Fe//D‐Phe diastereomers, respectively (Figure 3B), in good agreement with the experimental results. Comparison of the optimized geometries showed that the inversion of the chiral centers of the amino acids precludes the edge‐to‐face contacts [41]. Despite these are relatively weak interactions, here they work in a cooperative fashion (two from each Ph ring, six for each tripodal chiral inducer) with an additive effect in the stability (Figure 3B). Moreover, the helices of *mismatched* isomers slightly bend, as illustrated in the representations along the *C_3_* symmetry axes, which show a deviation from linearity of 5.7° for D‐Phe//Δ‐Fe//L‐Phe and 6.2° for D‐Phe//Δ‐Fe//D‐Phe (see Supporting Information).

**FIGURE 3:**
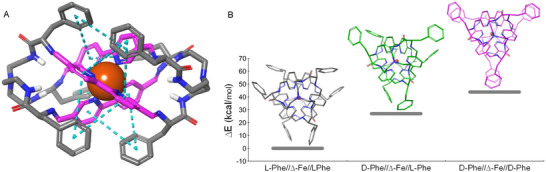
(A) DFT optimized structure (B3LYP‐D3 level of theory using the LACVP** basis set and PCM implicit solvation model for acetonitrile) for L‐**3**Fe displaying edge‐to‐face aromatic interactions (blue dashed lines) between Phe side chains and bipyridine moieties (highlighted in magenta). Non‐polar H atoms were omitted for clarity. (B) Plot of the relative energies (B3LYP‐D3/LACVP**, implicit acetonitrile) of the three possible combinations of relative chiral configurations (views along the *C_3_* symmetry axes are included).

Encouraged by these results, we wondered about the impact of freezing one exchange process in the chiral communication within the system. To that, we used a pre‐synthesized racemic Ru(II) octahedral complex, rac‐**4** in Figure 4 [42], which remains configurationally stable in solution at room temperature. An equilibrated mixture of L‐**1** and rac‐**4** showed a complex ^1^H NMR spectrum combining a set of sharp signals with *D_3_* symmetry in addition to extremely broad bands. These can be ascribed to the co‐existence of the corresponding Ru(II) cage with *matched* stereochemistry (namely L/Δ‐**3**Ru) and larger oligomers from the wrong chirality combination. Freezing the imine exchange by in situ reduction and subsequent purification allowed to isolate and fully characterize (NMR and MS, see Supporting Information) the Ru(II)‐polyamine cryptand L‐**5**Ru with a Δ‐Ru(bipy)_3_ configuration, as confirmed by CD spectroscopy (blue in Figure 4). The mass balance of the reaction suggested that the remaining Λ‐**4** reagent was trapped as polymeric byproducts that were impossible to isolate and characterize. The reaction also showed to be stereospecific, since the D‐**1** chiral inducer led to enantiomeric D/Λ‐**5**Ru (orange CD spectrum in Figure 4). In this case, since Ru(II)‐bipy coordination is unable to self‐correct, the dynamic imine exchange *selects* the right chirality of the metal complex in a helically ordered structure, discarding the wrong enantiomer as unproductive waste material.

**FIGURE 4:**
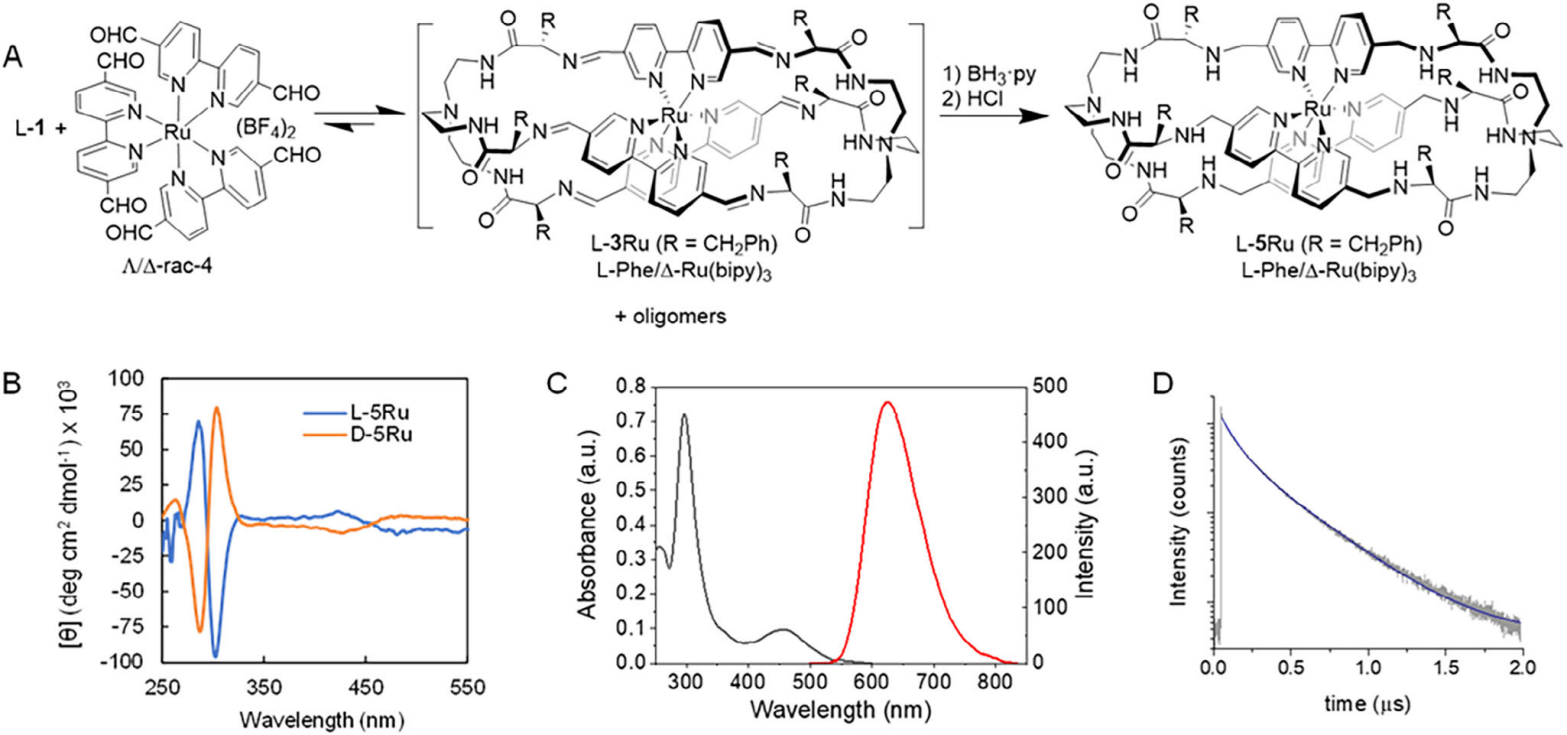
(A) Stereospecific dynamic selection of a helical Ru(II) pseudopeptidic cage. (B) CD spectra. (C) UV–visible absorption (black) and fluorescence emission spectra (red, *λ*_ex_ = 300 nm) of L‐**5**Ru in phosphate buffer, pH 7.3. (D) Photoluminescence decay trace (gray line) and biexponential decay fitting function (blue line) of L‐**5**Ru in phosphate buffer, pH 7.7 (*λ*_ex_ = 375 nm, *λ*_em_ = 600–660 nm).

Compound L‐**5**Ru was isolated as the corresponding HCl salt with very good aqueous solubility over a wide pH range. Typically, Ru(II) metal complexes usually exhibit interesting photophysical properties, luminescence emission, and potential applications as photosensitizers and photocatalysts, so we investigated the properties of compound L‐**5**Ru in aqueous solution. L‐**5**Ru exhibited an MLCT absorption band at 480 nm and the typical, very intense LC absorption of bipy groups at 285 nm (Figure 4C). The emission spectrum showed a broad band centered at 623 nm, with a photoluminescence quantum yield of (0.8 ± 0.1)%. Photoluminescence decay traces showed a bi‐exponential behavior, with an average photoluminescence lifetime of 330 ± 20 ns (Figure 4D and Supporting Information). Both the quantum yield and lifetime showed negligible dependence on the pH of the solution in the range 3.0–9.0 (see Supporting Information). In addition, we investigated whether L‐**5**Ru exhibited circularly polarized light (CPL) emission; however, the low quantum yield of the molecule prevented to obtain conclusive results. The high solubility of these cages in water is an advantage compared to other Ru(II) complexes in the literature, allowing potential applications as cell imaging agents. Moreover, the long luminescence lifetime of the cage, arising from a forbidden ^3^MLCT triplet → singlet transition, makes it especially interesting for advanced microscopy techniques, such as fluorescence lifetime imaging (FLIM), and as a photosensitizer in photoinduced upconversion and photodynamic therapy. Future biological applications are envisioned for these chiral pseudopeptidic metal cryptates, with the possibility of external decoration and bioconjugation thanks to their modular assembling.

## Conflicts of Interest

The authors declare no conflict of interest.

## Supporting information




**Supporting File 1**: Full characterization data (NMR, MS, XRD), control experiments, and molecular modeling details are included in the electronic Supporting Information [1–7].

**Supporting File 2**: anie71315‐sup‐0002‐SuppMat.pdb.

## Data Availability

The data that support the findings of this study are available in the Supporting Information of this article.
