# Supplementary material for: Stereospecific Assembly of Triply Chiral Pseudopeptidic Cages Through Dynamic Dual Chirality Transfer
Source: Angew Chem Int Ed Engl. 2026 Jan 28;65(11):e25964. doi: 10.1002/anie.202525964 (PMC12970502; doi:10.1002/anie.202525964)
Supplement: Supplementary file 1 — Supporting File 1: Full characterization data (NMR, MS, XRD), control experiments, and molecular modeling details are included in the electronic Supporting Information [1–7]. [file ANIE-65-e25964-s001.pdf]

SUPPORTING INFORMATION

FOR

Stereospecific Assembly of Triply Chiral Pseudopeptidic Cages Through Dynamic Dual Chirality Transfer

Tushar D. Bhosale,[a] Eudald Usan,[a] Delia Miguel,[b] Jordi Solà,[a] Ángel Orte,[b] Ciril Jimeno,[*,a] and Ignacio Alfonso[*,a]

[a] T. D. Bhosale, E. Usan, Dr. J. Solà, Dr. C. Jimeno, Prof. I. Alfonso
Department of Biological Chemistry
Institute for Advanced Chemistry of Catalonia, IQAC-CSIC
Jordi Girona 18-26, 08034, Barcelona (Spain)
E-mail: ciril.jimeno@iqac.csic.es; ignacio.alfonso@iqac.csic.es

[b[Dr. D. Miguel, Prof. A. Orte
Nanoscopy-UGR Laboratory, Departamento de Fisicoquímica,
Facultad de Farmacia, Unidad de Excelencia de Química,
University of Granada, 18071, Granada (Spain)

<u>CONTENTS</u>

0. MATERIALS AND METHODS S2
1. IRON CAGE **3**Fe SYNTHESIS PROCEDURE AND CHARACTERIZATION DATA S4
2. RUTHENIUM CAGE **5**Ru SYNTHESIS PROCEDURE AND CHARACTERIZATION DATA S11
3. IMINE CAGE **3** SYNTHESIS PROCEDURE AND CHARACTERIZATION DATA S18
4. SCRAMBLING EXPERIMENTS INVOLVING **3**Fe CAGE S21
5. XRD STRUCTURE AND CRYSTALLOGRAPHIC DATA OF **3**Fe S27
6. THEORETICAL CALCULATIONS ON **Fe-3** S30
7. SPECTROPHOTOMETRIC EXPERIMENTS WITH **Ru-5** AT DIFFERENT pH S35
8. REFERENCES S36

0. MATERIALS AND METHODS

Reagents and solvents were purchased from commercial suppliers (Aldrich, TCI or Merck) and were used without further purification. All the compounds prepared were fully characterized by the complete NMR spectroscopic data and mass spectrometry (MALDI-TOF).

Preparative reverse phase purifications were performed on an Isolera Biotage instrument (KP C18-HS column, CH_3CN and water with 0.1% TFA as mobile phases). Analytical RP-HPLC was performed with a Hewlett Packard Series 1100 (UV detector 1315A) modular system using a reverse-phase Kromasil 100 C8 (15 x 0.46 cm, 5 μm) column. CH_3CN-H_2O mixtures containing 0.1% TFA at 1 mL/min were used as mobile phase and monitoring wavelengths were set at 220, 254 and 280 nm.

NMR spectroscopy: The NMR experiments were carried out at 25˚C on a VNMRS-400 NMR spectrometer (Agilent Technologies 400 MHz for ^{1}H and 100 MHz for ^{13}C for characterization. Chemical shifts are reported in ppm using the solvent residual peak as a reference. Data were processed with the software program MNova (Mestrelab Research).

MALDI-TOF mass spectrometry: Performed on a Bruker Autoflex III smartbeam spectrometer. All samples were prepared using α-cyano-*p*-hydroxycinnamic acid as matrix.

CD and UV-Vis spectroscopy: CD and UV-Vis spectra were simultaneously measured in solution in a standard UV-Vis quartz cubette of 1 cm optical pathway. A Jasco J-1500 spectrometer under N_2 flow thermostatized at 20 °C using a JASCO CTU-100 circulation thermostat unit was used.

UV-visible spectroscopy measurements were made at 25 °C on a Cary 60 UV-vis spectrophotometer, using a 0.5 cm path-length quartz cell and 4x10^{-5}M solutions of Ru-cage complex in 10mM phosphate buffered solutions. Steady-state fluorescence emission spectra were obtained on a Jasco FP-8300 Spectrofluorometer (Jasco, Tokyo, Japan). PL emission quantum yields were obtained using the relative method, with fluorescein in aqueous NaOH 0.1 M as the reference. Quantum yields were obtained by averaging results from two different concentrations of sample, two concentrations of reference, and two different excitation wavelengths.

Time-resolved fluorescence emission and the collection of PL decay traces was carried out on a MicroTime 200 (PicoQuant GmbH) system, based on an Olympus IX71 microscope. The samples of L-**5** in aqueous phosphate buffer at different pH values were transferred to a multi-well chambered coverglass (Ibidi) and placed in the microscope, equipped with an oil-immersion, 1.4NA, $\times$100 objective. The excitation source was a 375-nm pulsed laser (PicoQuant) set at a repetition rate of 0.5 MHz using a Sepia II driver (PicoQuant). The excitation laser was focused 10 μm inside the solution and PL detection was collected through the same objective, filtered by a 405-nm longpass filter and focused on a 100 μm confocal aperture. Then, PL emission was detected on a hybrid PMT (PicoQuant), after passing a 630/60 nm bandpass filter, mainly centered on the L-**5** complex emission band. Photon time-tagging was performed on a MultiHarp 150 module (PicoQuant). Ten different decay traces were collected for each sample. These traces were globally fitted to biexponential decay functions using SymphoTime 64 (PicoQuant) and the intensity-weighted average lifetime was reported.

Crystal structure determination of **CJM881ALT** was carried out using a Rigaku diffractometer equipped with a Pilatus 200K area detector, a Rigaku MicroMax-007HF microfocus rotating anode with MoK_α radiation, Confocal Max Flux optics and an Oxford Cryosystems low temperature device Cryostream 700 plus (T = -173 ˚C). Full-sphere data collection was used with ω and φ scans. *Programs used:* Data collection and reduction with CrysAlisPro V/.60A (Data collection and reduction with CrysAlisPro 1.171.42.96, Rigaku OD, 2023) and absorption correction with Scale3 Abspack scaling algorithm

(Empirical absorption corrrection using spherical harmonics implemented in Scale3 Abspack scaling algorithm, CrysAlisPro 1.171.42.96, Rigaku OD, 2023) Structure Solution and Refinement: Crystal structure solution was achieved using the computer program SHELXT.[1] Visualization and processing of the structure was performed with the program Olex2.[2] Missing atoms were subsequently located from difference Fourier synthesis and added to the atom list. Least-squares refinement on F^2 using all measured intensities was carried out using the program SHELXL 2015.[3] All non-hydrogen atoms were refined including anisotropic displacement parameters. Comments to the structure: CJM881ALT: The asymmetric unit contains one molecule of the cationic iron complex, two chlorine anions, two BF4-anions, 1.75 molecules of acetonitrile and 0.4 molecules of water. The main molecule is partly disordered in two orientations (chain C35-45 ratio 77:23 and chain C5-C11 ratio 67:33). The acetonitrile molecules are disordered in 8 positions (ratio: 0.5:0.25:0.2:0.2:0.2:0.15:0.15:0.10). The water molecule is also located in the area of disordered solvent molecules with an occupancy of 0.4.

Molecular modeling was performed with Jaguar[4] as implemented in Maestro 14.2 from Schrödinger.[5] Starting from the X-ray diffraction experimental geometry, we performed a Monte Carlo conformational Search with OPLS4 force field[6] to confirm that the geometry was stable and that HCl molecules can be safely removed from the simulations. Global minimum was fully optimized at the DFT B3LYP-D3 level of theory and with the LACVP** basis set,[7] using implicit acetonitrile PCM solvation model. For the generation of the corresponding diastereomers in Figure 3 of the manuscript, we manually inverted three or six phenylalanine chiral centers and the minima were obtained following the same procedure and level of theory.

1. IRON CAGE **3**Fe SYNTHESIS PROCEDURE AND CHARACTERIZATION DATA

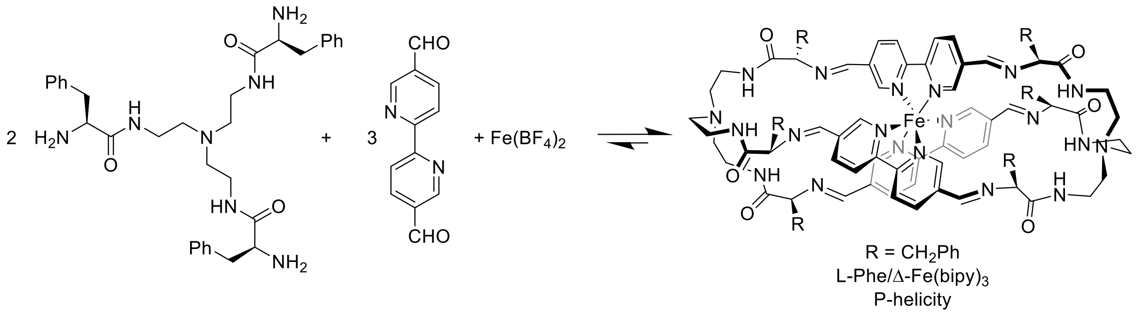

Scheme S1.1

Synthetic procedure: 2,2'-bipyridine-4,4'-dicarbaldehyde (8.2 mg, 0.0386 mmol) was weighed into a flask and dissolved in 2 ml of CD_3CN. Then, iron(II) tetrafluoroborate hexahydrate (4.3 mg, 0.0129 mmol) dissolved in 2 ml of CD_3CN was added. After 15 minutes, the reaction mixture was further diluted with 4 ml of CD_3CN and then the tren-phenylalanine ligand (15.2 mg, 0.0258 mmol) dissolved in 1 ml of $CDCl_3$ was added dropwise. The mixture was stirred at room temperature for 24 hours.

NMR data for **3**Fe:

^{1}H-NMR ($CD_3CN/CDCl_3$ 8/1): δ 8.71 (d, *J*=8 Hz, 1H), 8.61 (d, *J*=2 Hz, 1H), 8.26 (broad dxd, J^1=9.2 Hz, J^2=4 Hz, 1H), 8.09 (dxd, J^1=8 Hz, J^2=2 Hz, 1H), 7.67 (s, 1H), 7.06 (t, *J*=7.6 Hz, 1H), 6.93 (t, *J*=7.6 Hz, 2H), 7.76 (d, *J*=6.8 Hz, 2H), 3.90 (dxd, J^1=10 Hz, J^2=3.2 Hz, 1H), 3.86 (m, 1H), 3.23 (dxd, J^1=13.2 Hz, J^2=3.2 Hz, 1H), 3.10 (m, 1H), 2.84 (m, 1H), 2.69 (dxd, J^1=13.2 Hz, J^2=10.4 Hz, 1H), 2.63 (m, 1H) ppm.

^{13}C NMR (100 MHz, CD_3CN) δ 172.4 (C), 159.4 δ 172.4 (C), 159.43 (C), 158.9 (CH), 156.5 (CH), 140.3 (CH), 138.5 (C), 133.6 (C), 130.5 (CH), 128.92 (CH), 127.5 (CH), 124.2 (CH), 76.2 (CH), 61.5 (CH_2), 41.6 (CH_2), 35.0 (CH_2) ppm.

Full ^{1}H-NMR spectrum for **3**Fe:

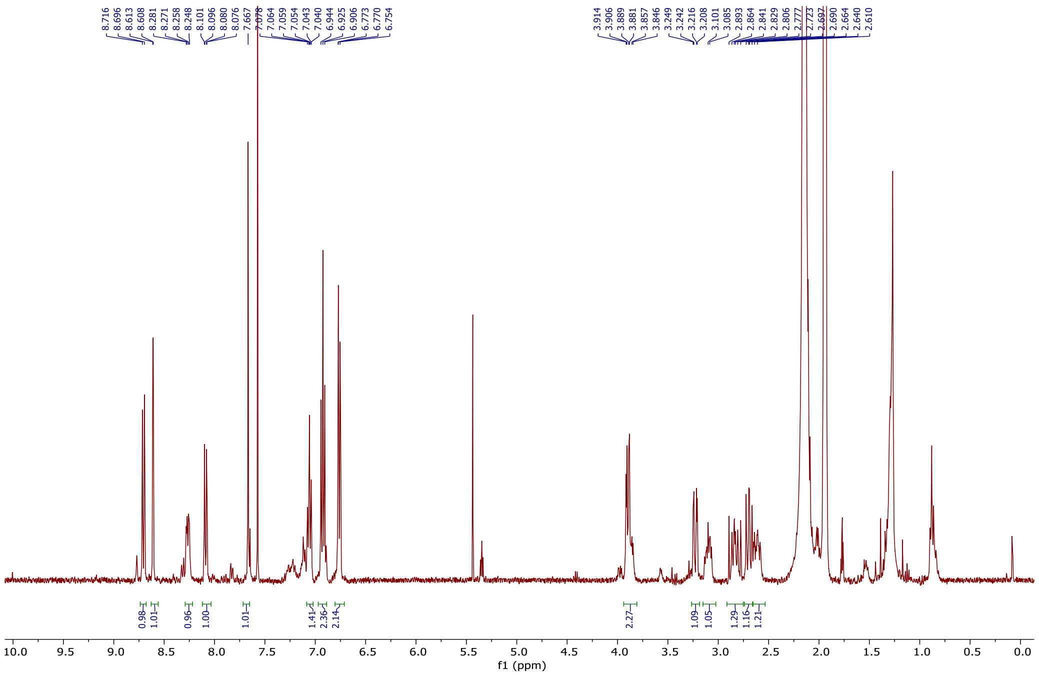

Figure S1.1

Aromatic region expansion for **3**Fe:

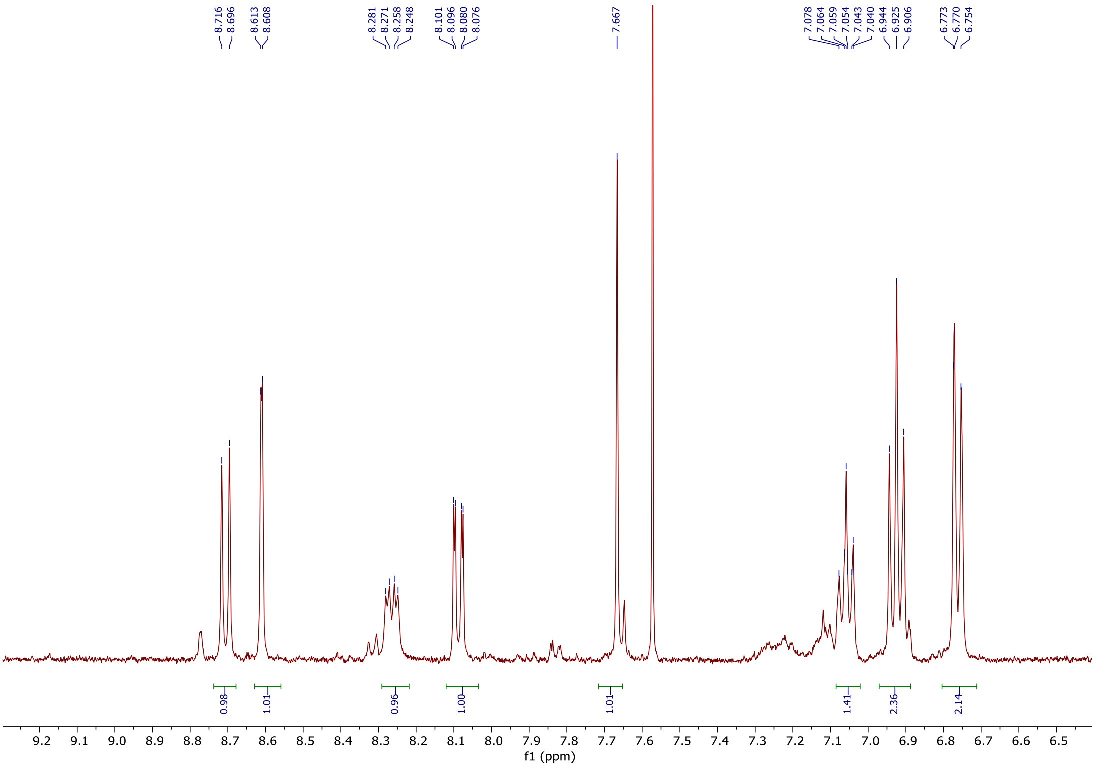

Figure S1.2

Aliphatic region expansion for **3**Fe:

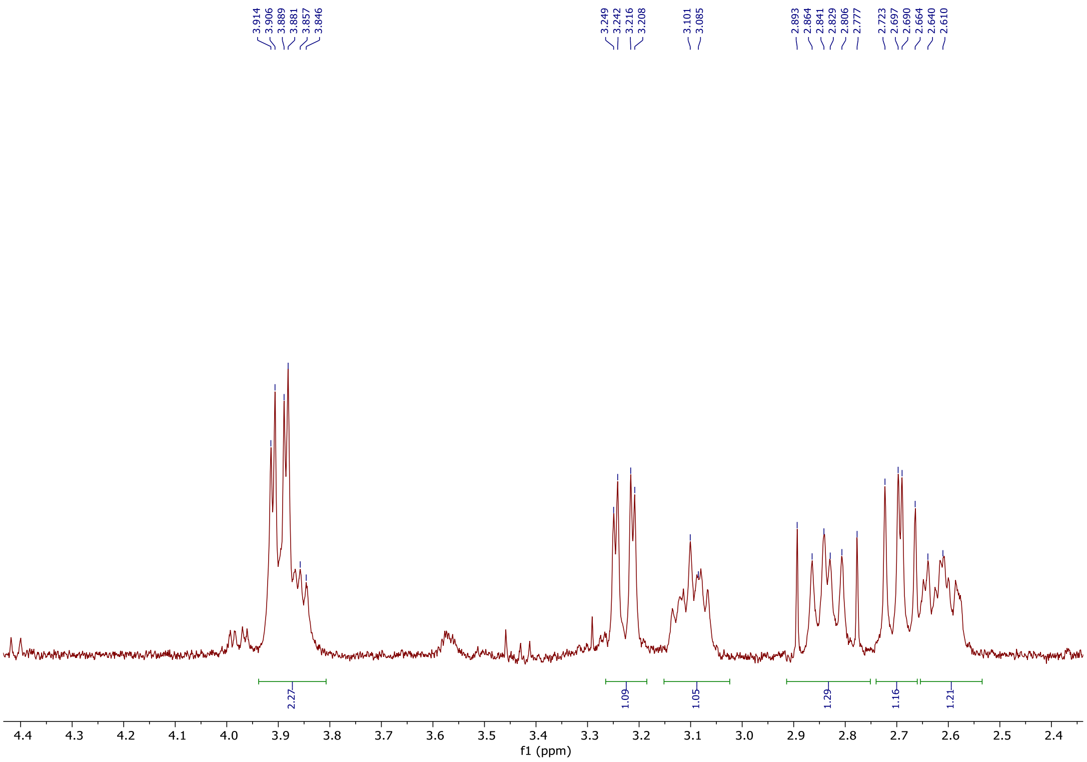

Figure S1.3

^{13}C NMR (APT, CD_3CN, 100 MHz) for **3**Fe:

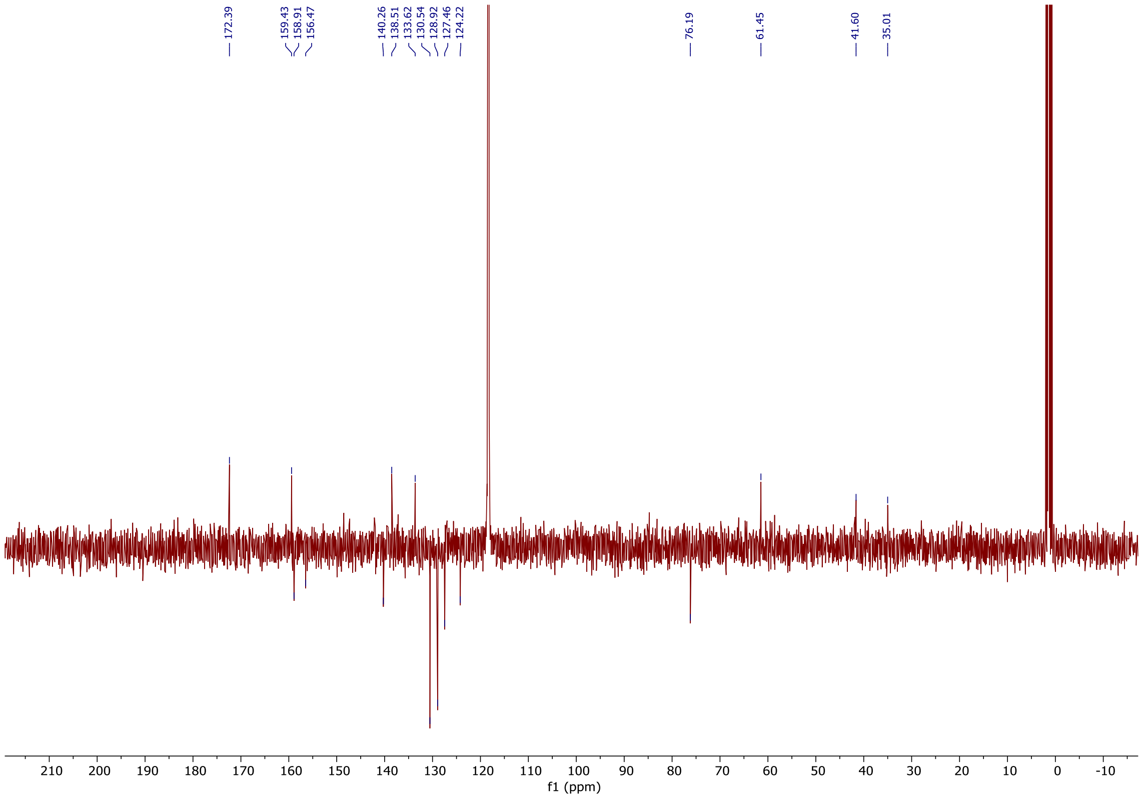

Figure S1.4

COSY (CD_3CN) for **3**Fe:

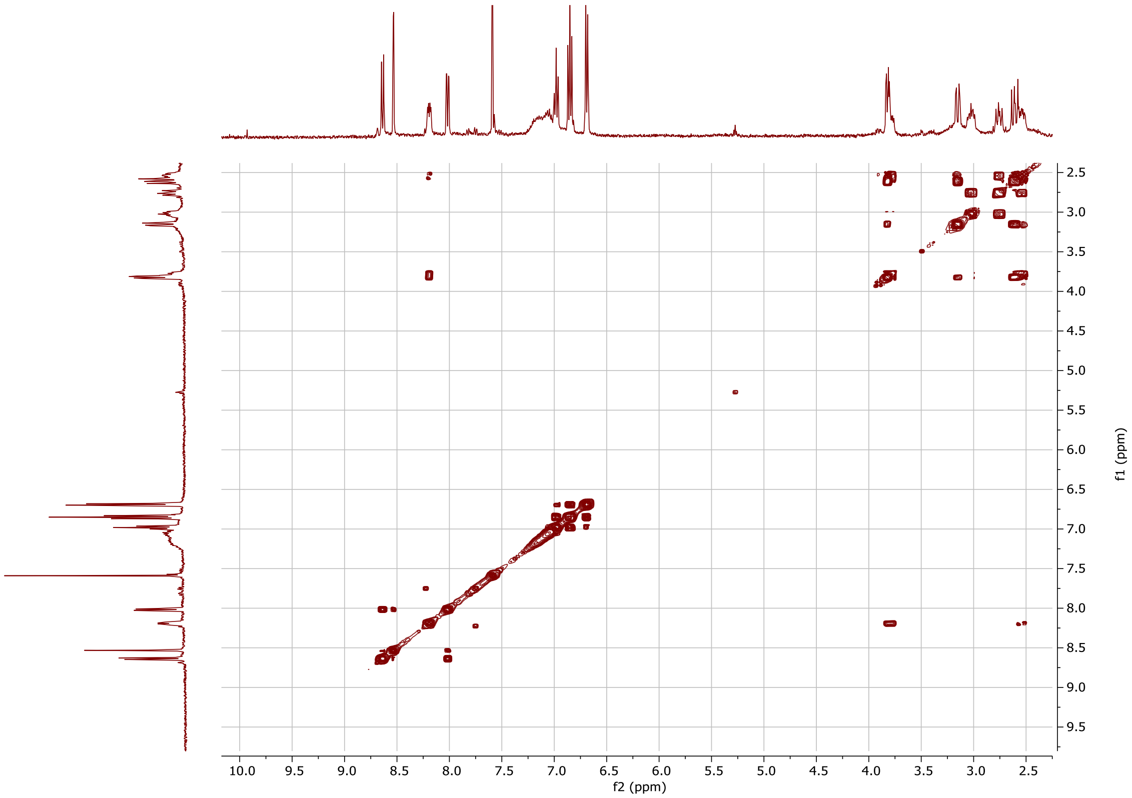

Figure S1.5

COSY expansions for **3**Fe:

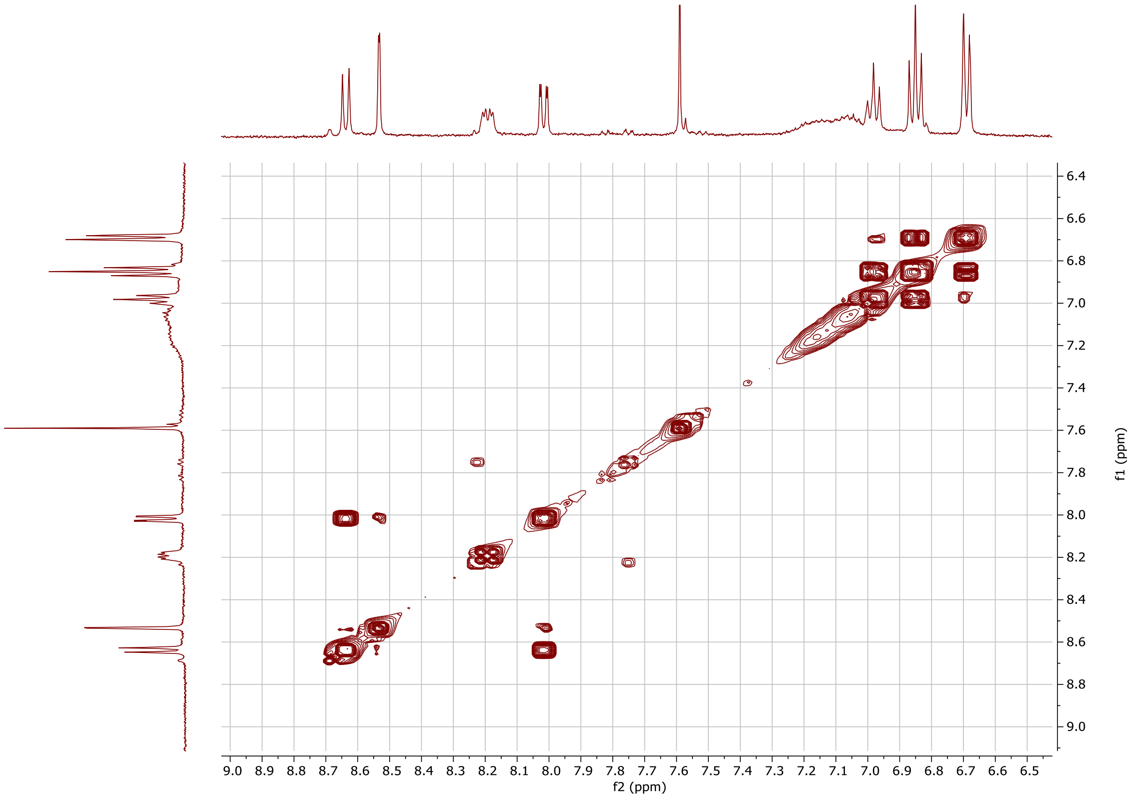

Figure S1.6

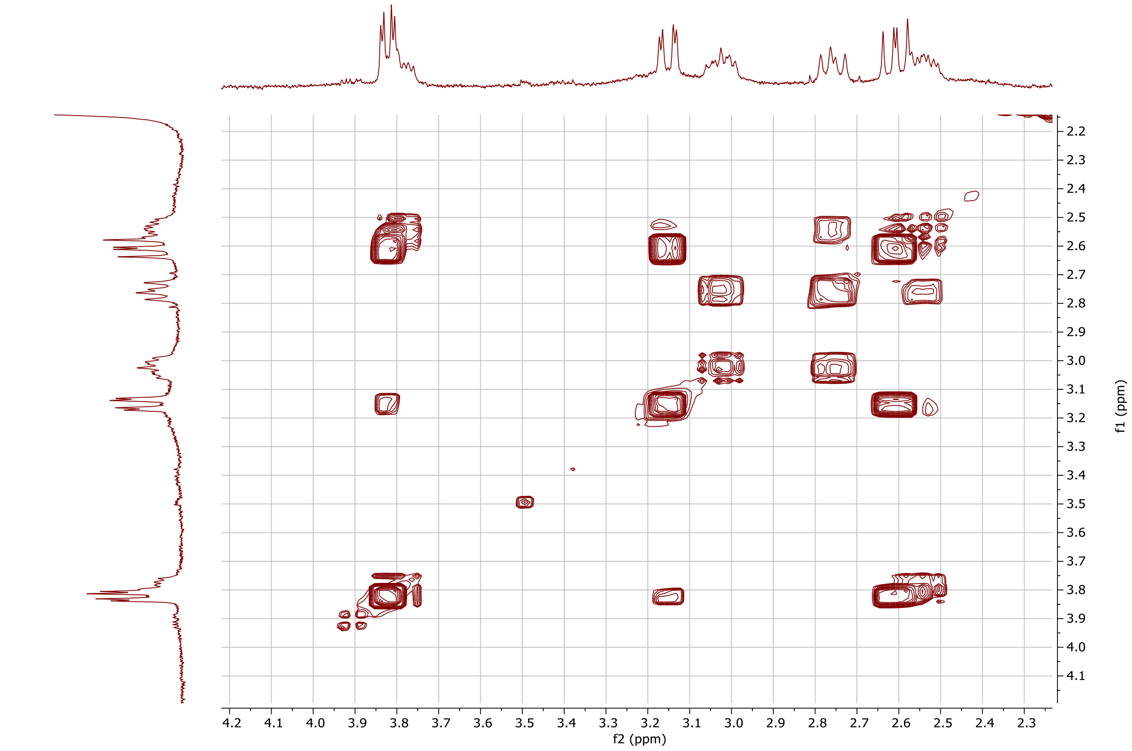

Figure S1.7

HSQC for **3**Fe:

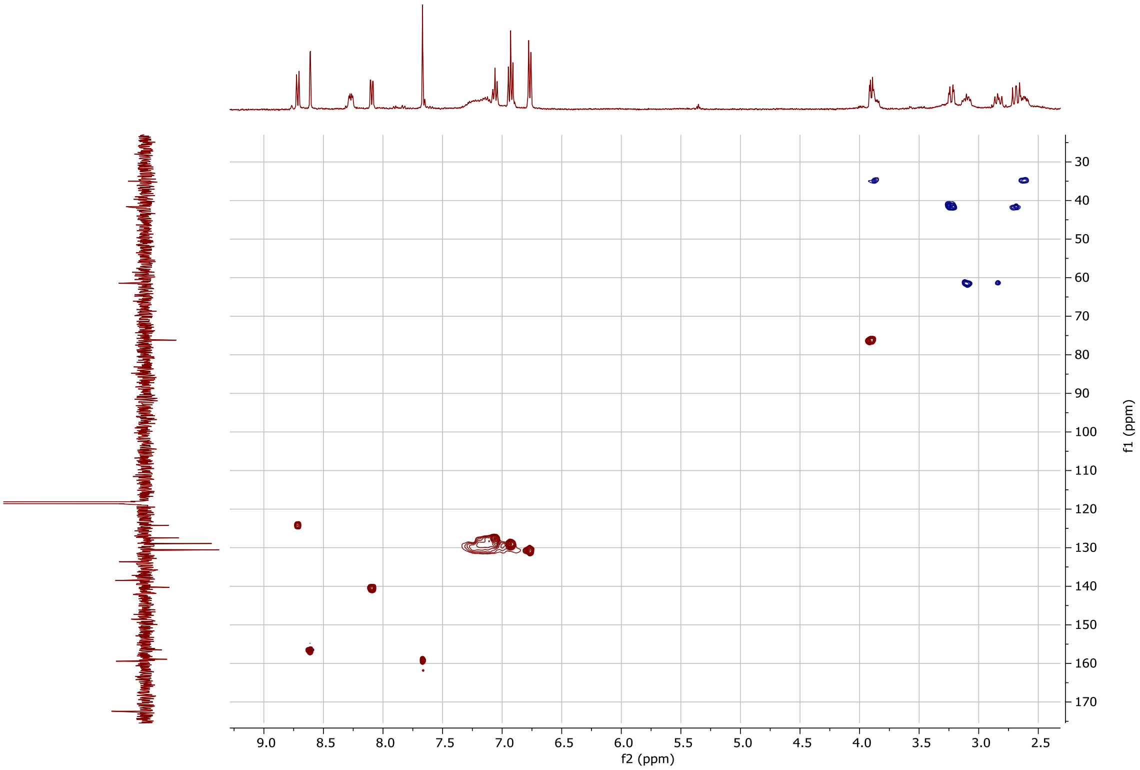

Figure S1.8

ROESY for **3**Fe:

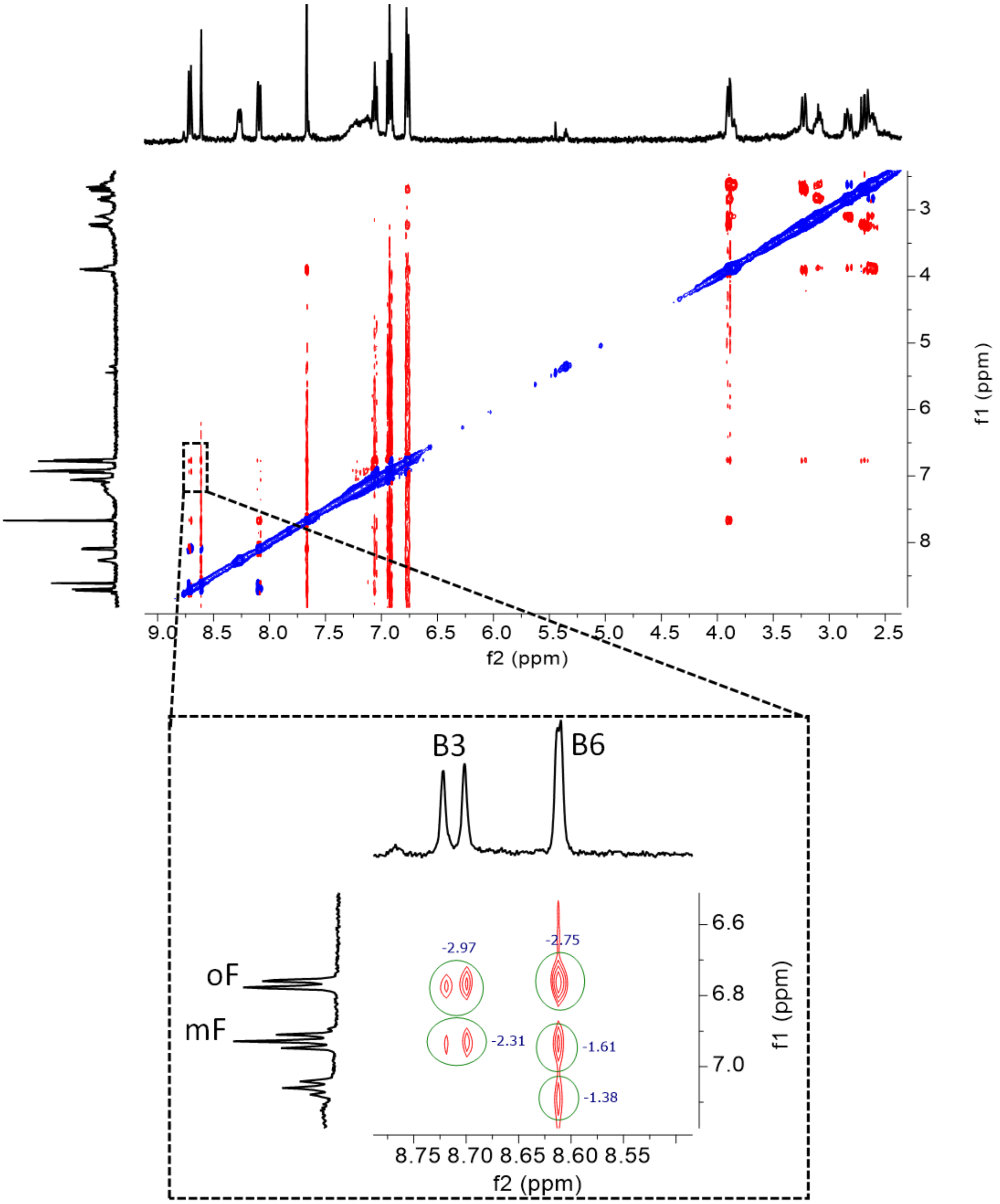

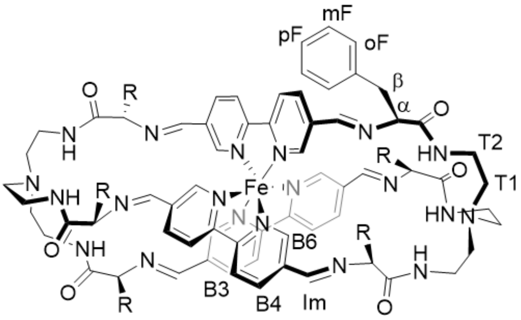

d (Å)	oF	mF
B3	2.95	3.54
B6	2.56	4.06

Figure S1.9. Existing NOE contacts between bipyridine and phenyl protons are shown. Contact distances are calculated (See the Theoretical Calculations section below).

MS data for **3**Fe:

MS: top, simulated spectrum for C102H101N20O6Fe; bottom, MALDI-TOF spectrum for **Fe-3**.

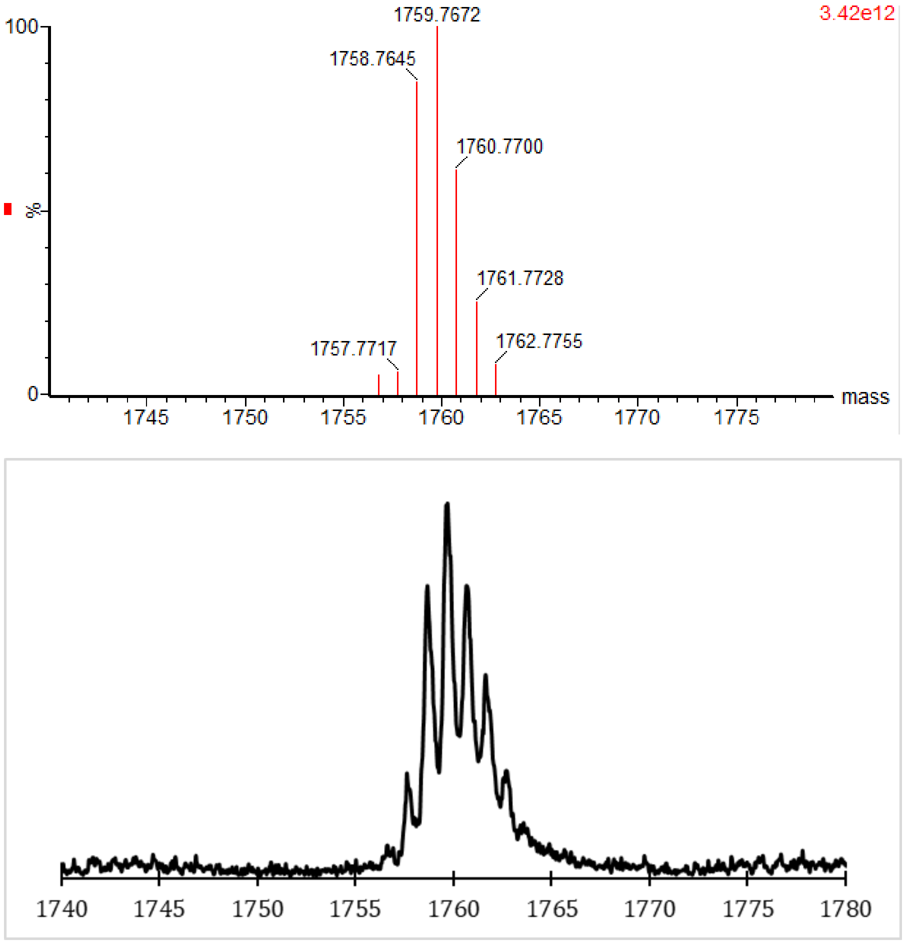

Figure S1.10

UV-Vis at 0.021 mM in $CHCl_3$ for **3**Fe:

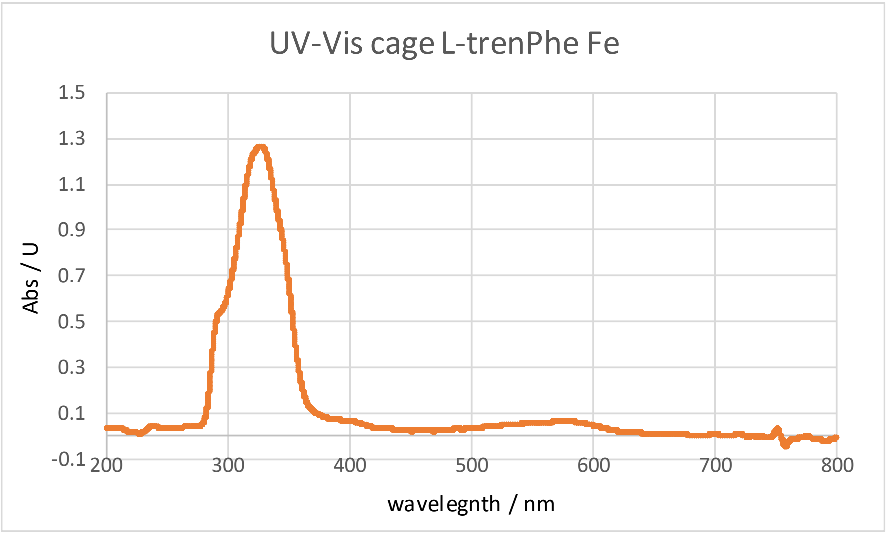

Figure 1.11

2. RUTHENIUM CAGE **5**Ru SYNTHESIS PROCEDURE AND CHARACTERIZATION DATA.

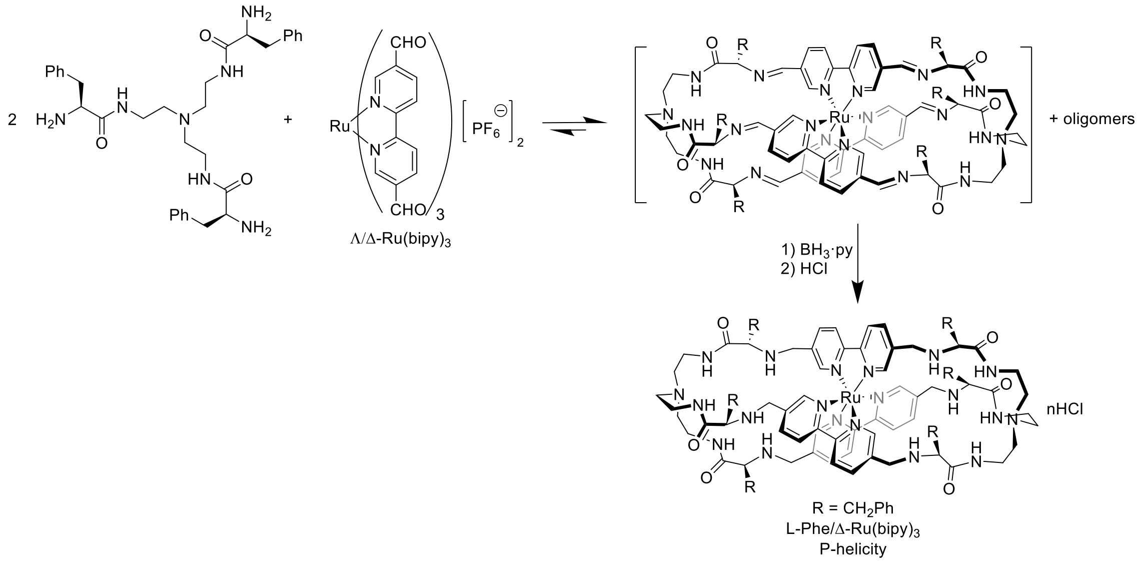

Scheme 2.1

Synthetic procedure:

Ruthenium(II) tris(2,2’-bipyridine-4,4’-dicarbandehyde) hexafluorophosphate (10.3 mg, 0.01 mmol) were dissolved in 5 ml of acetonitrile. L-trenPhe ligand (11.8 mg, 0.02 mmol) dissolved in 5 ml of acetonitrile were added dropwise, and the mixture was stirred at room temperature for 24 hours. After ^{1}H NMR checking that no starting aldehyde was left in the reaction mixture, 20 μL of 8 M pyridine·borane THF solution (0.16 mmol) were added and left reacting overnight. Then, the reaction was quenched with 5 mL of 1 M HCl and subsequently all solvents were removed in the rotavap. The residue was purified by automatic chromatography on C18-silica gel eluting with acetonitrile:water from 5% to 100% acetonitrile in 30 minutes at 15 mL/min. The fraction thus collected was freeze dried, affording 13 mg of the Ru(II) cage (69% yield).

NMR data for **5**Ru:

^{1}H NMR (400 MHz, D_2O) δ 8.53 (d, J = 8.4 Hz, 1H), 8.18 – 7.92 (m, 1H), 7.71 (d, J = 1.8 Hz, 1H), 7.48-7.37 (m, 3H), 7.35 – 7.26 (m, 2H), 4.53 (s, 2H), 4.25 (t, J = 7.6 Hz, 1H), 3.45 (m, 2H), 3.32 – 3.13 (m, 2H), 3.05 (m, 2H) ppm.

^{13}C NMR (100 MHz, D_2O) δ 169.8 (C), 156.0 (C), 149.2 (CH), 140.2 (C), 136.1 (CH), 133.8 (C), 129.4 (CH), 129.2 (CH), 128.1 (CH), 123.8 (CH), 60.2 (CH_2), 54.4 (CH), 51.6 (CH_2), 36.8 (CH_2), 33.8 (CH_2) ppm.

Full ^{1}H-NMR spectrum for **5**Ru::

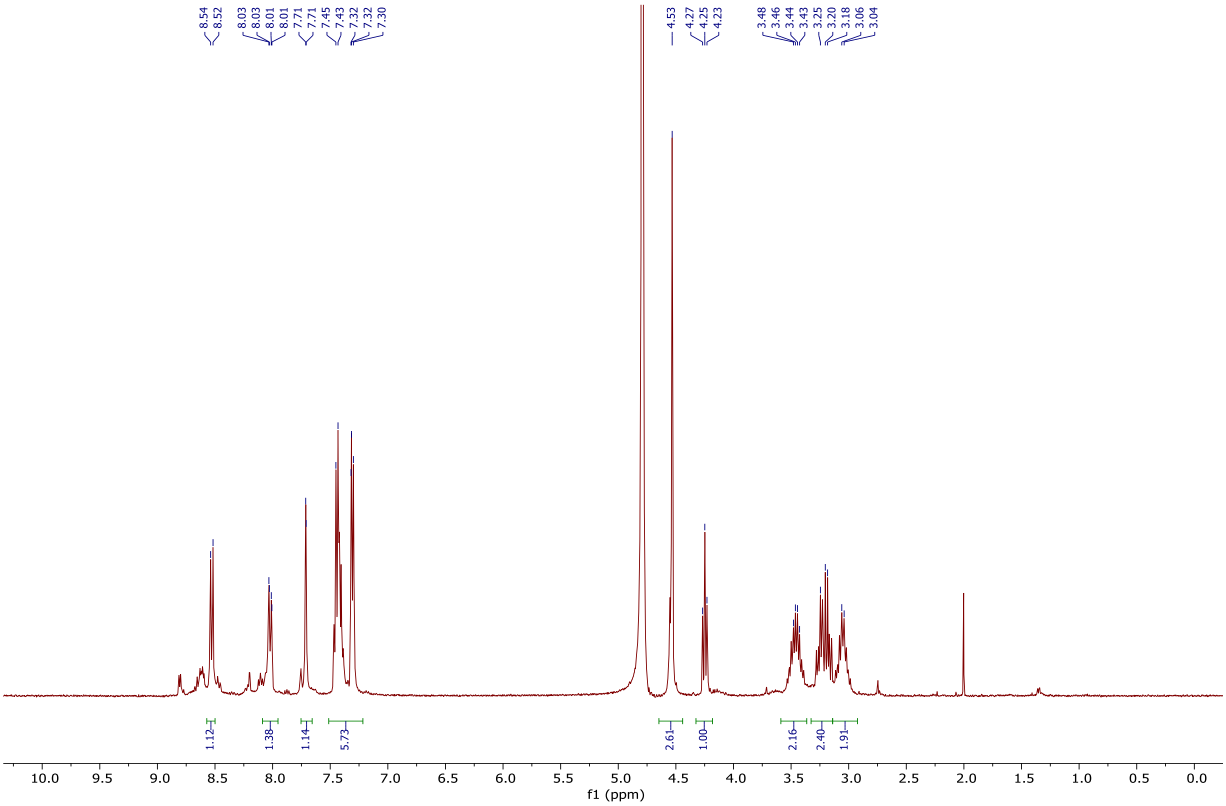

Figure S2.1

Aromatic region expansion for **5**Ru::

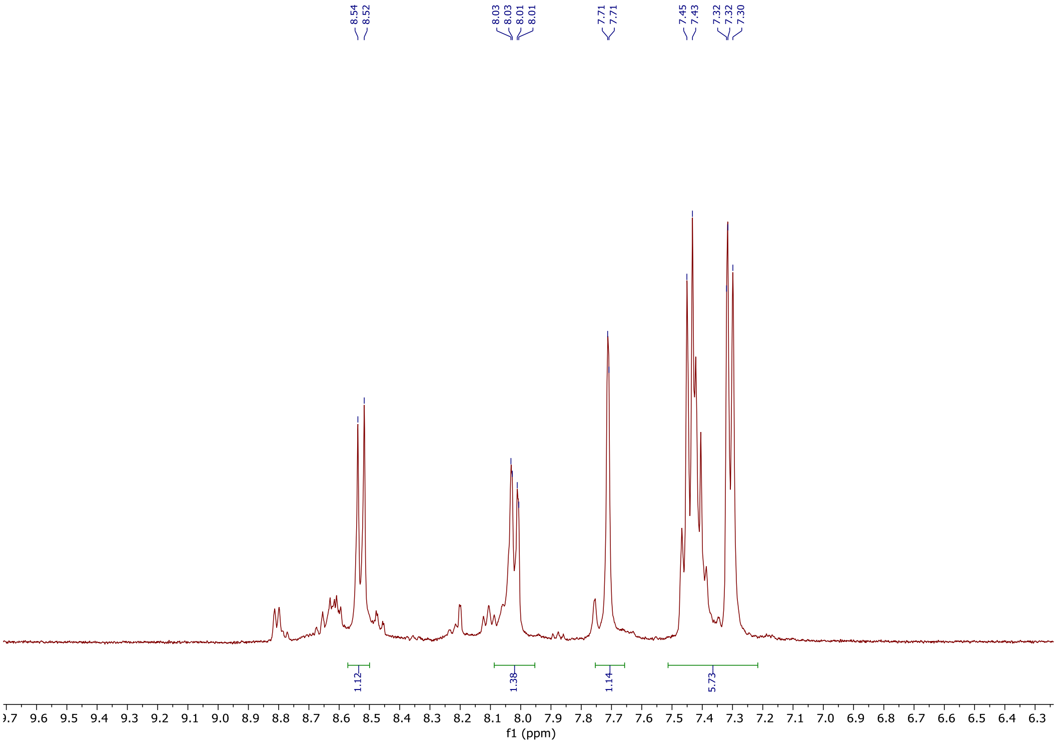

Figure S2.2

Aliphatic region expansion for **5**Ru::

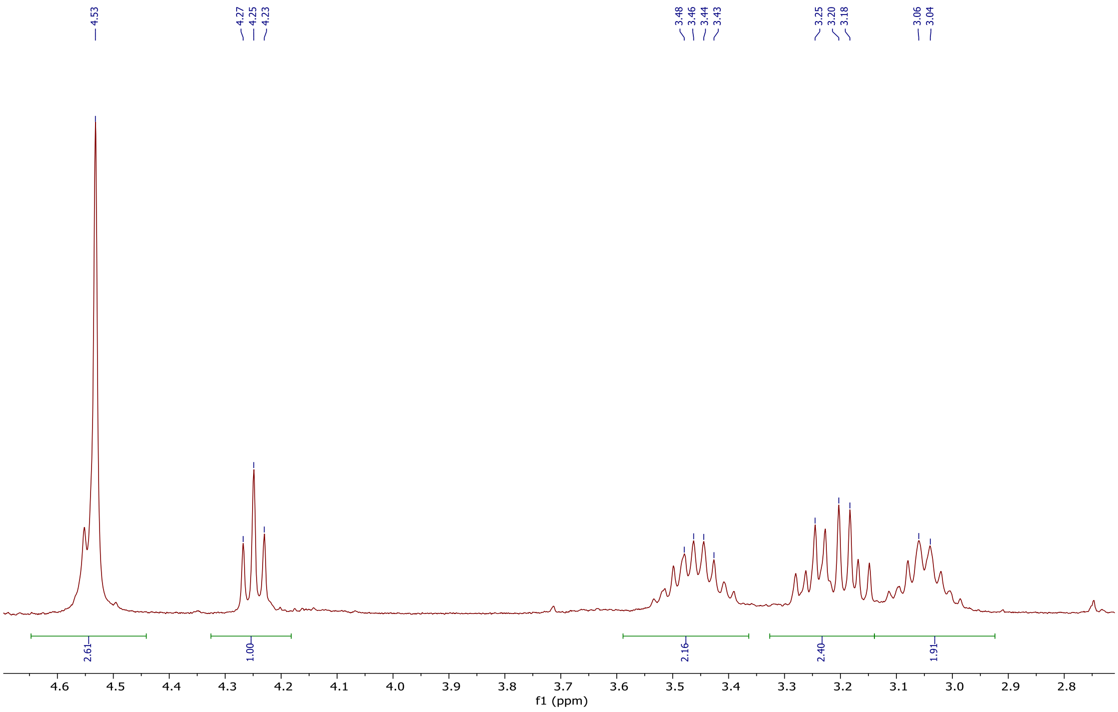

Figure S2.3

^{13}C NMR (APT, D_2O, 100 MHz) full spectrum and aromatic region expansion for **Ru-5**::

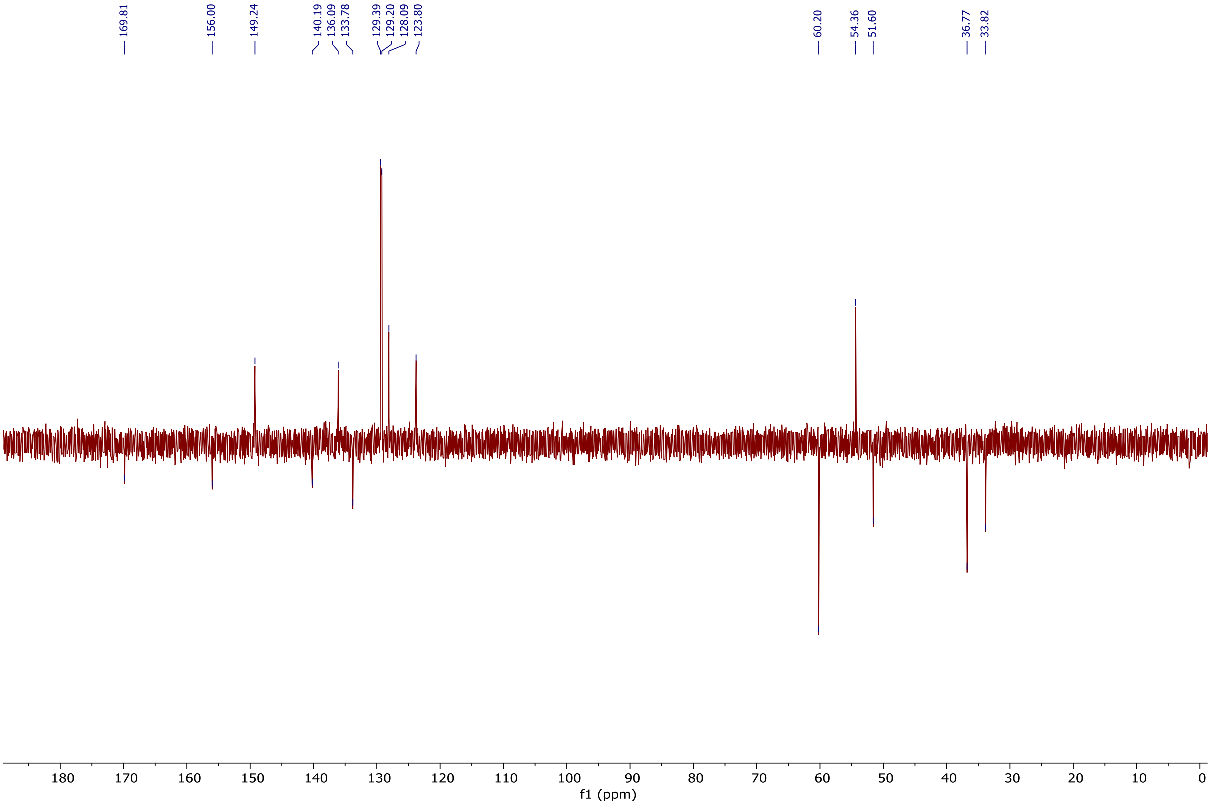

Figure S2.4

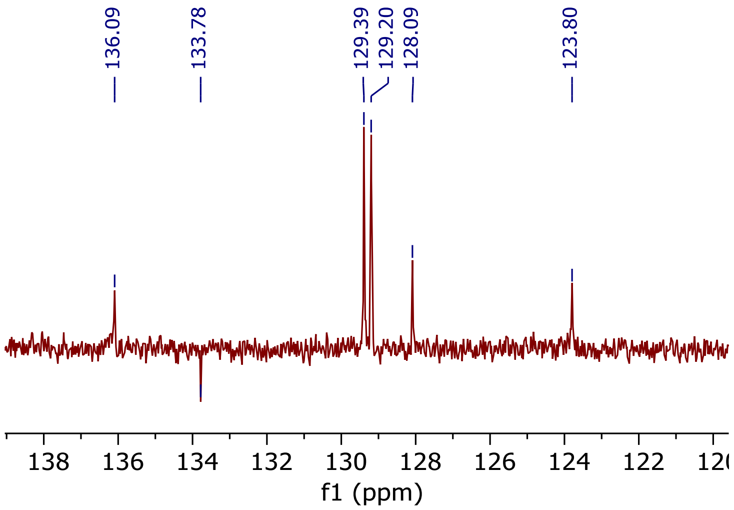

Figure S2.4 (expanded area)

COSY, full spectrum and expansions for **5**Ru:

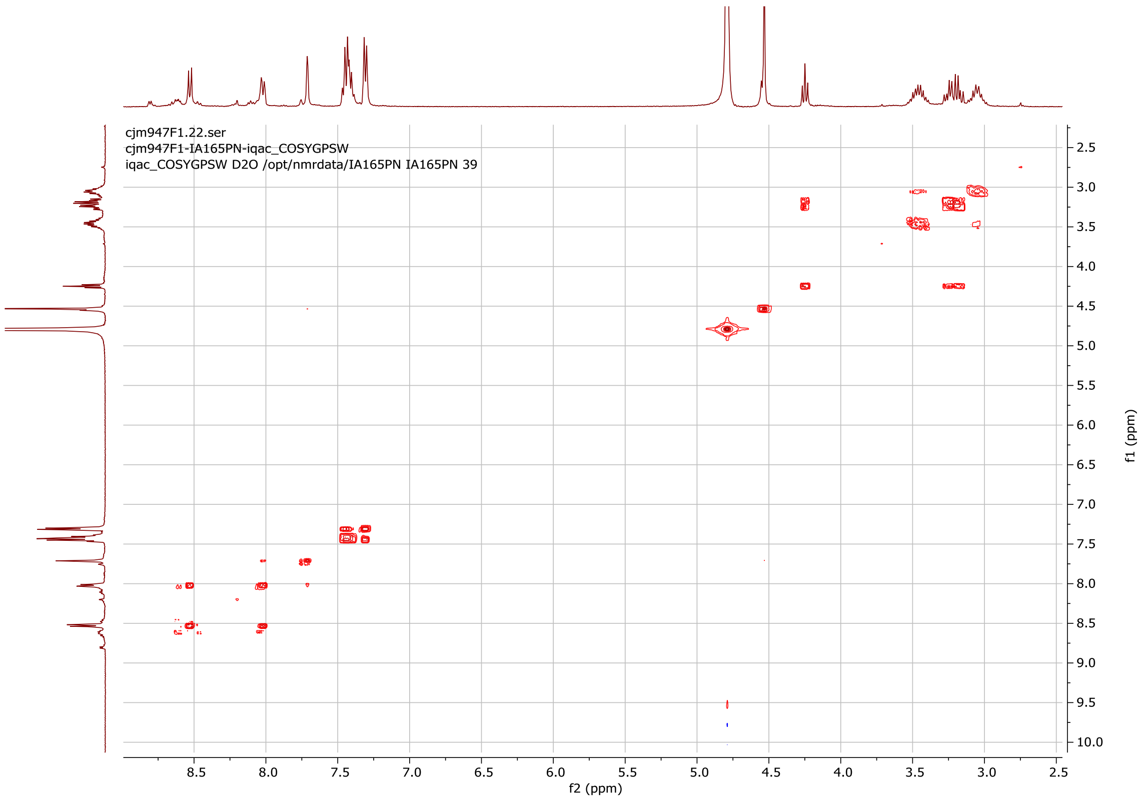

Figure S2.5

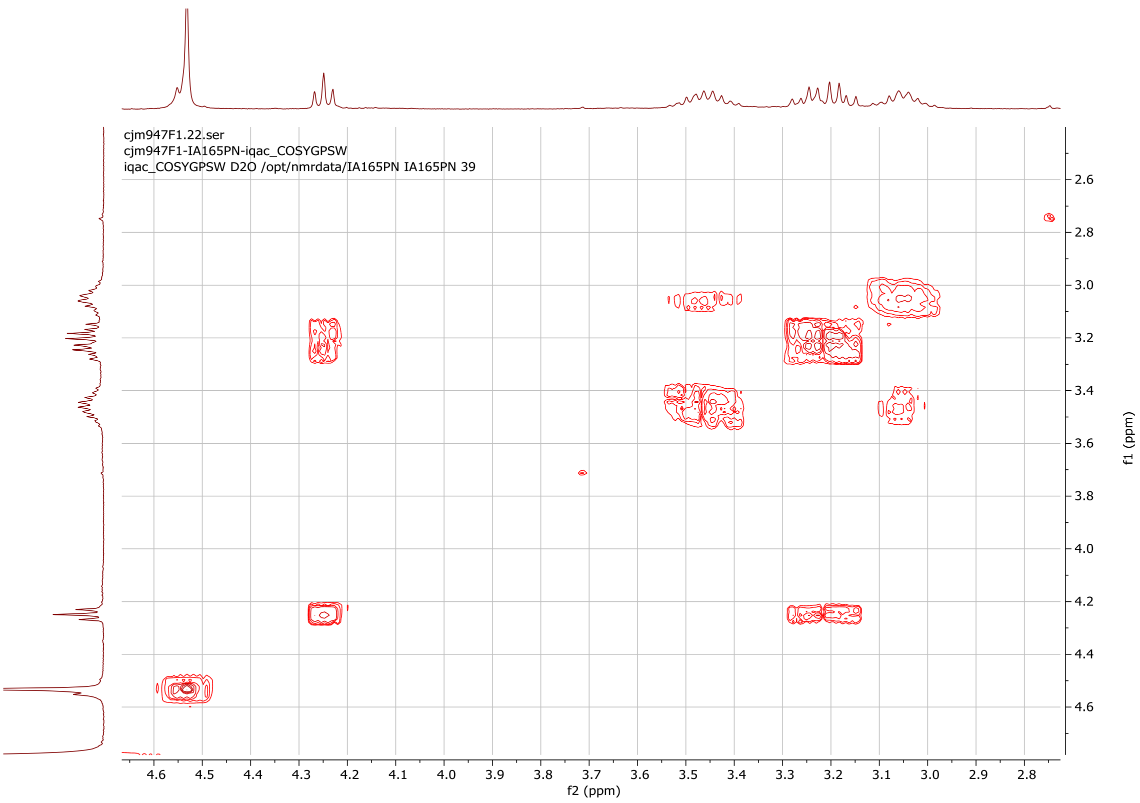

Figure S2.6

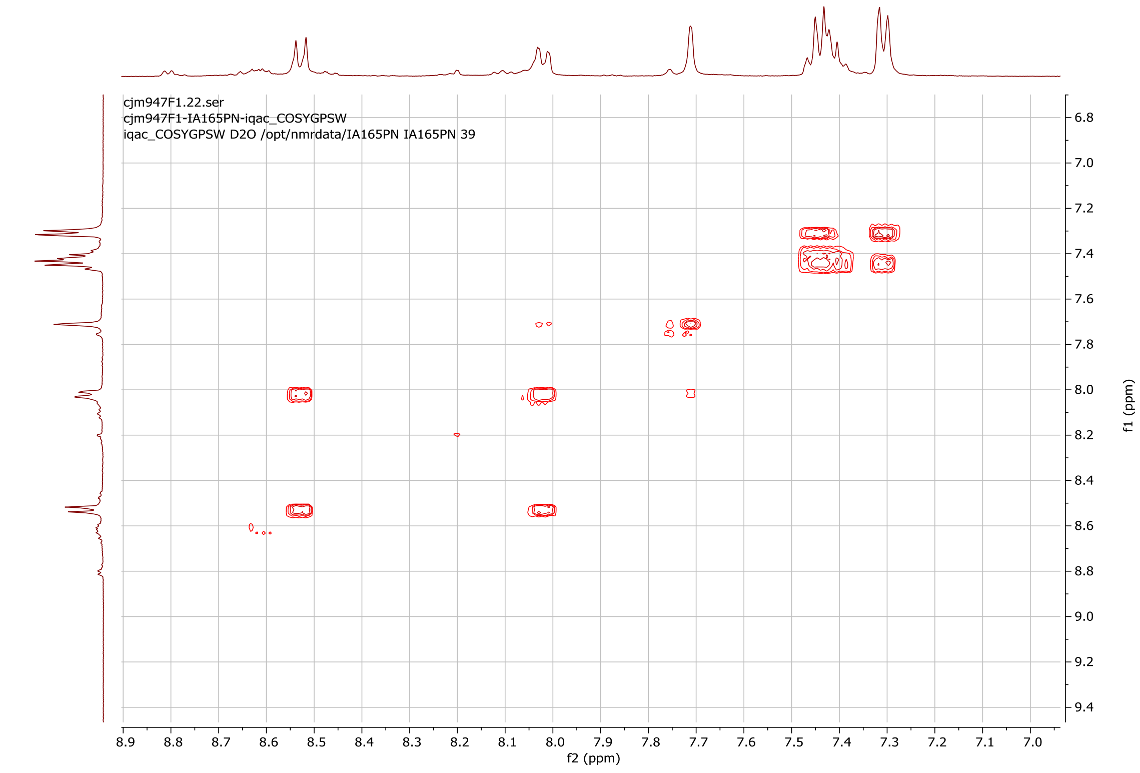

Figure S2.7

HSQC, full spectrum for **5**Ru::

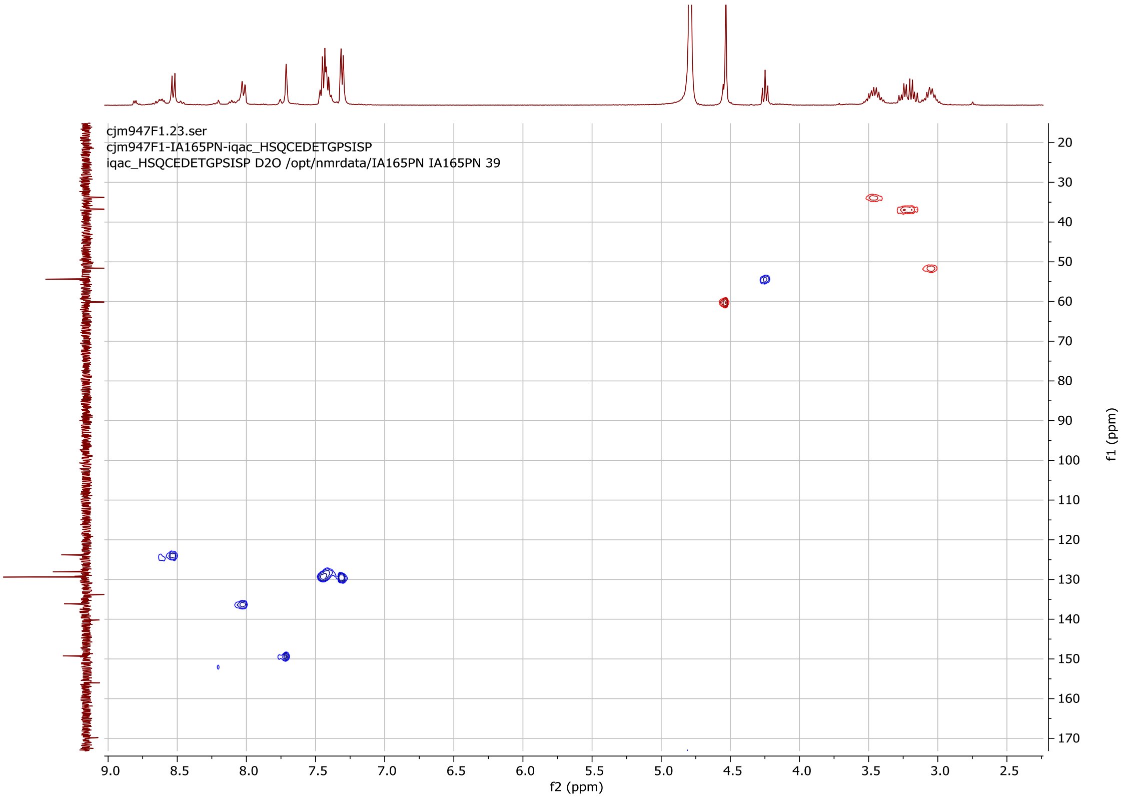

Figure S2.8

MS data for for **5**Ru:

Ru cage: top, simulated spectrum for $C_{102}H_{114}N_{20}O_6Ru$; bottom, MALDI-TOF spectrum.

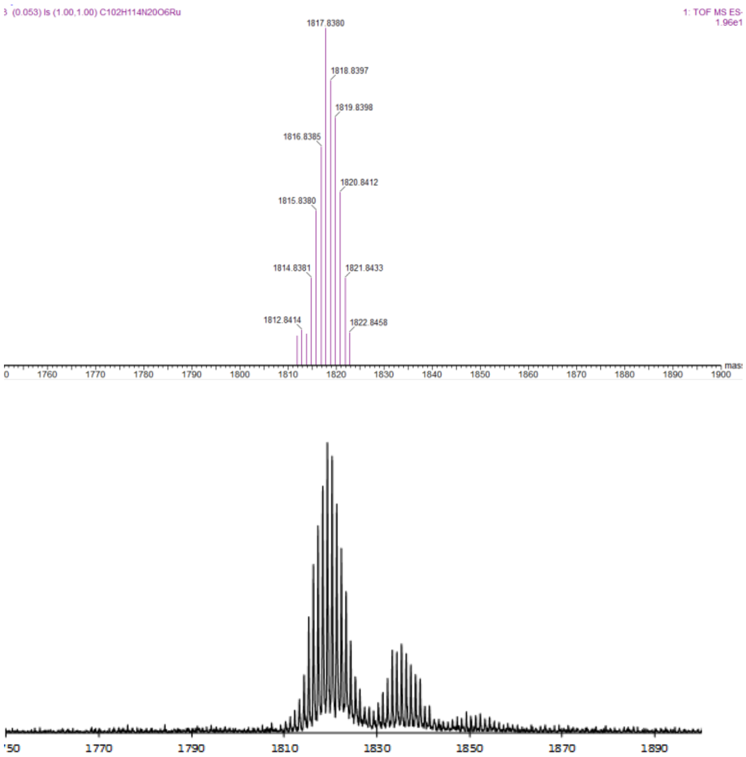

Figure S2.9

UV-Vis stectrum for for **5**Ru:

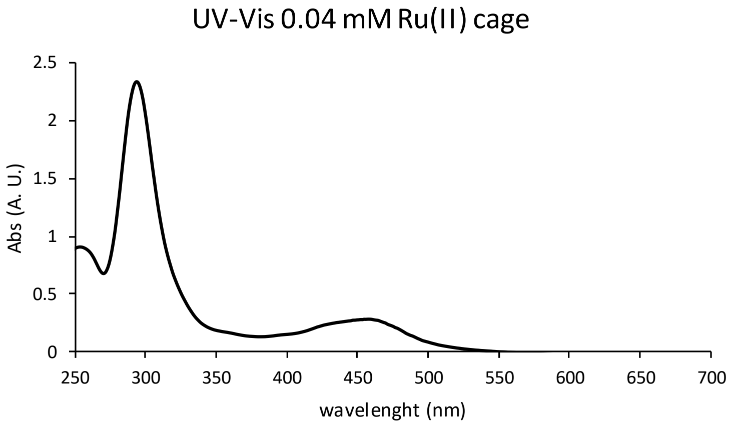

Figure S2.10

3. IMINE **3** CAGE SYNTHESIS PROCEDURE AND CHARACTERIZATION DATA

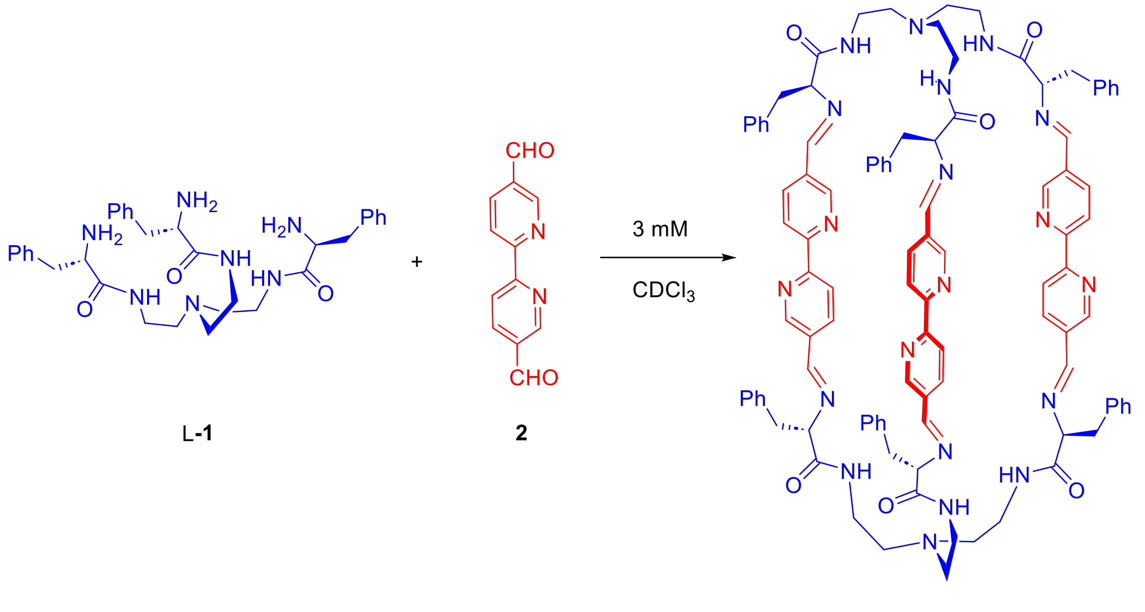

Scheme 3.1

Synthetic procedure:

2,2’-bipyridine-4,4’-dicarbaldehyde **2** (3.82 mg, 0.018 mmol) were weighed into a flask and dissolved in 0.8 mL of $CDCl_3$. Then, 200 µL of 60 mM tren-phenylalanine ligand L-**1** (0.012 mmol) in $CDCl_3$ were added. The mixture was stirred at room temperature for 24 h and analyzed directly by NMR.

NMR data for **3**:

^{1}H-NMR (400 MHz, $CDCl_3$): δ 8.78 (d, *J* = 2.1 Hz, 1H), 8.48 (d, *J* = 8.2 Hz, 1H), 7.94 (dd, *J* = 8.2, 2.2 Hz, 1H), 7.53 (s, 1H), 7.14 – 7.05 (m, 3H), 6.94 (dd, *J* = 7.6, 1.9 Hz, 2H), 3.98 (dd, *J* = 10.1, 3.2 Hz, 1H), 3.59 – 3.44 (m, 1H), 3.38 (dd, *J* = 13.6, 3.2 Hz, 1H), 3.32 – 3.21 (m, 1H), 2.78 (m, 1H), 2.71 (dd, *J* = 13.5, 10.1 Hz, 1H) ppm.

^{13}C NMR (101 MHz, $CDCl_3$) δ 171.8 (C), 159.6 (CH), 157.1 (C), 149.5 (CH), 137.29 (C), 136.1 (CH), 131.1 (C), 130.1 (CH), 128.4 (CH), 126.8 (CH), 121.5 (CH), 75.5 (CH), 53.3 (CH_2), 41.1 (CH_2), 37.6 (CH_2) ppm.

^{1}H-NMR (400 MHz, $CDCl_3$) for **3**:

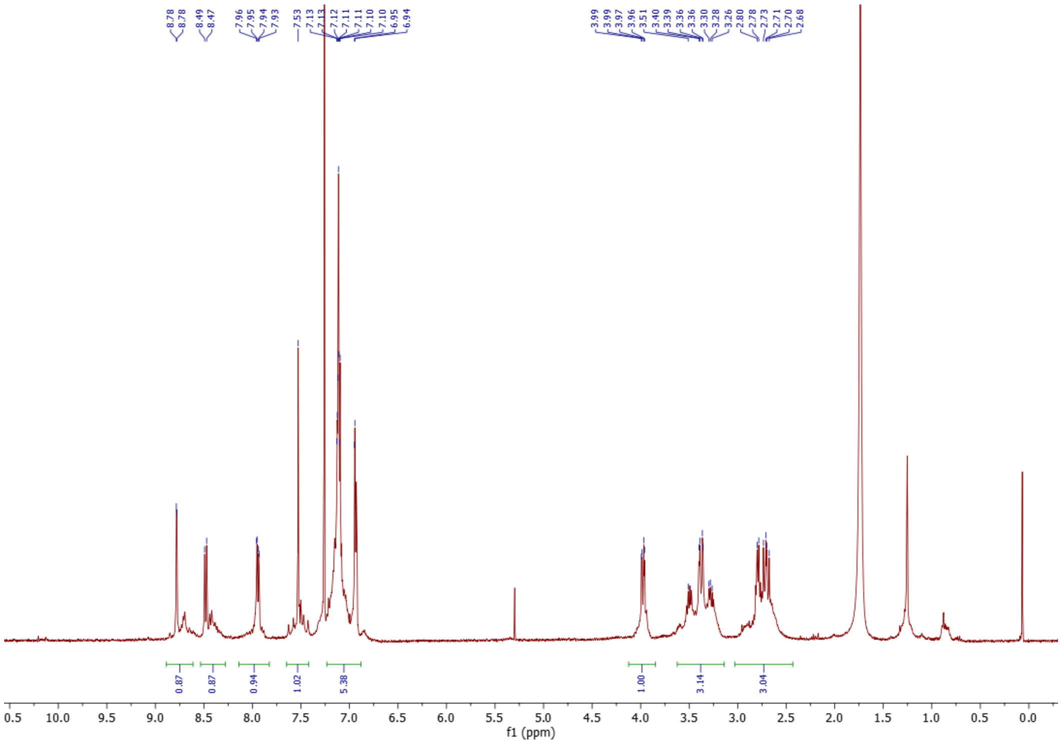

Figure 3.1

COSY (400 MHz, $CDCl_3$) for **3**:

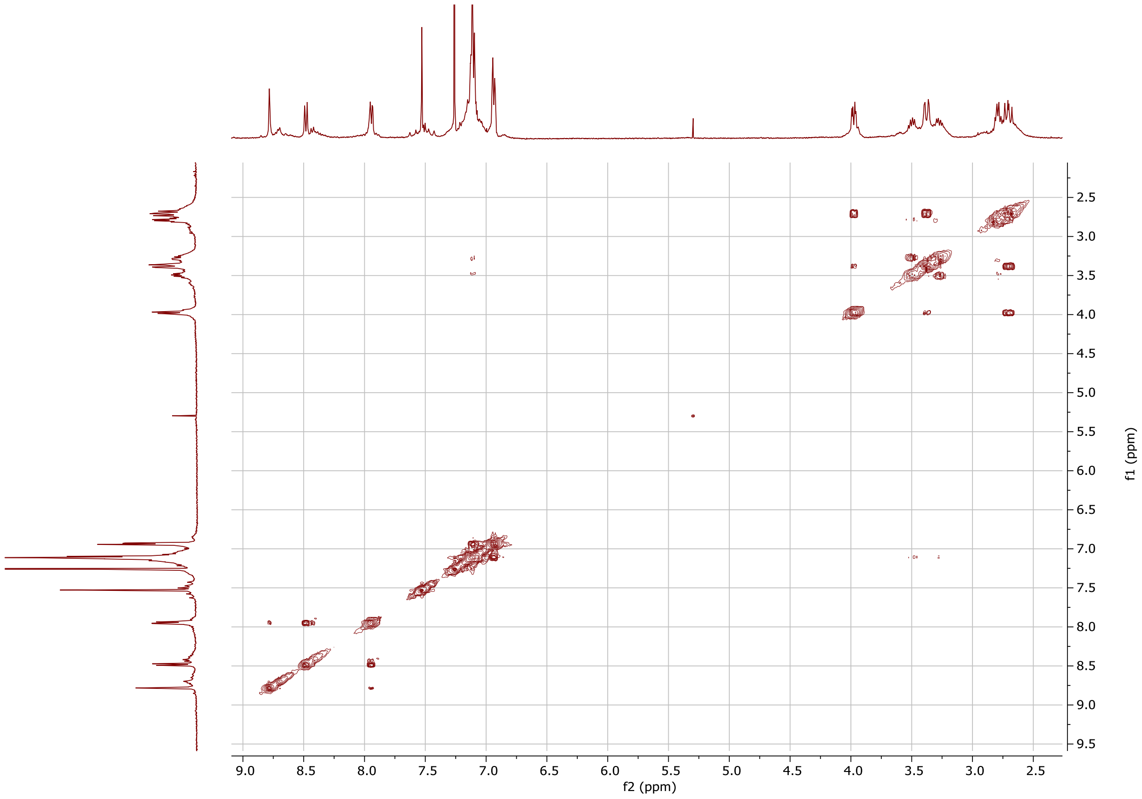

Figure S3.2

HSQC for **3**:

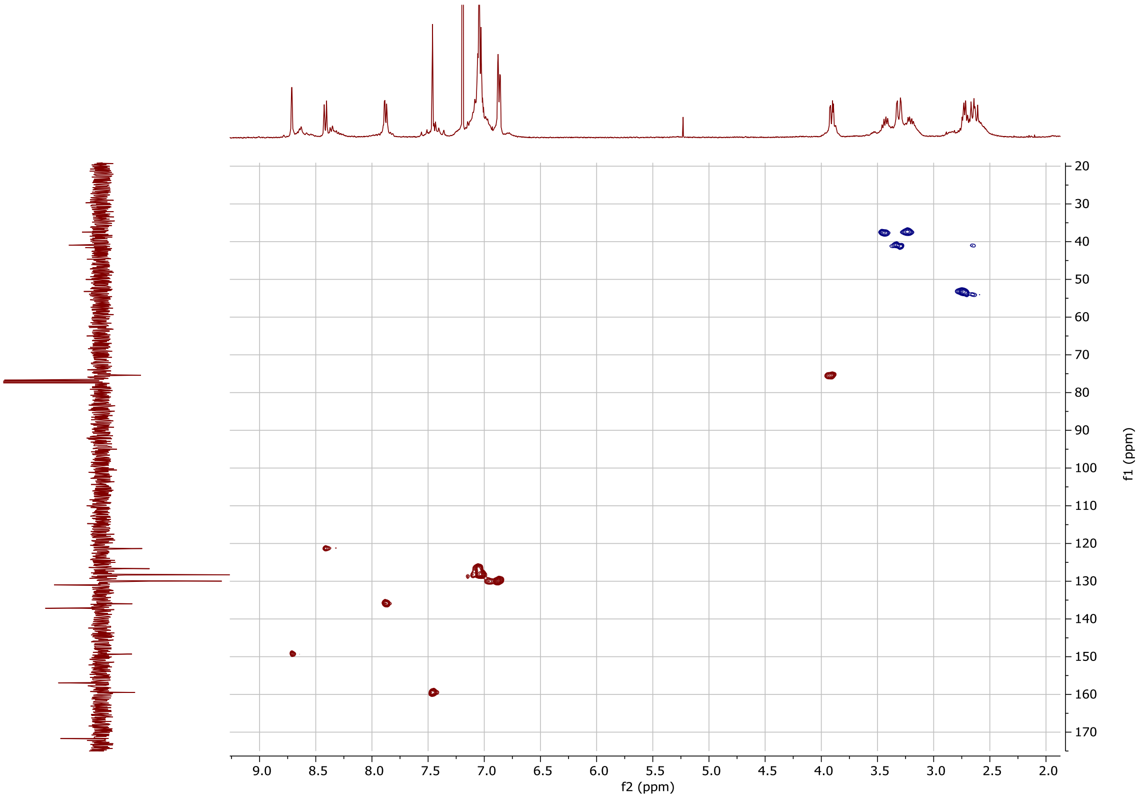

Figure S3.3

UV-Vis spectrum of **3.**

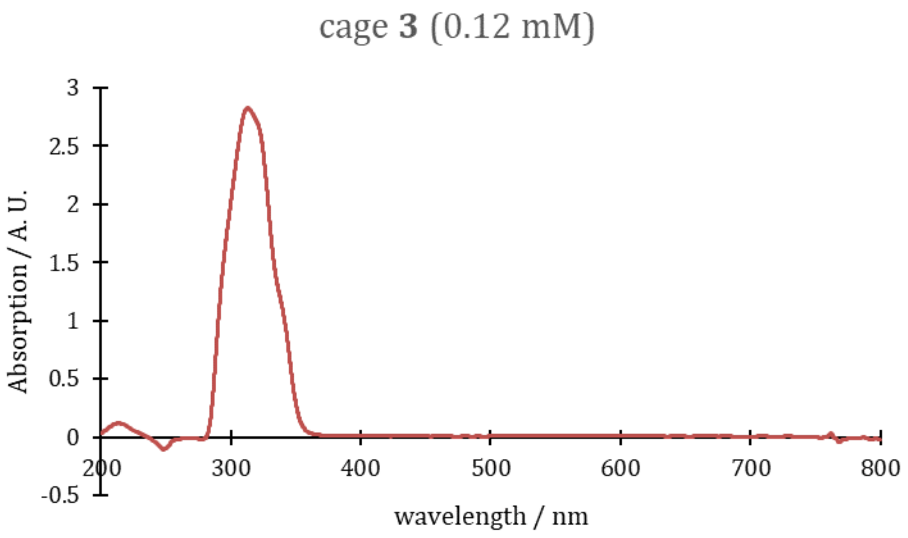

Figure S3.4

4. <u>SCRAMBLING EXPERIMENTS INVOLVING Fe CAGE **3**:</u>

MALDI-TOF from reaction mixtures.

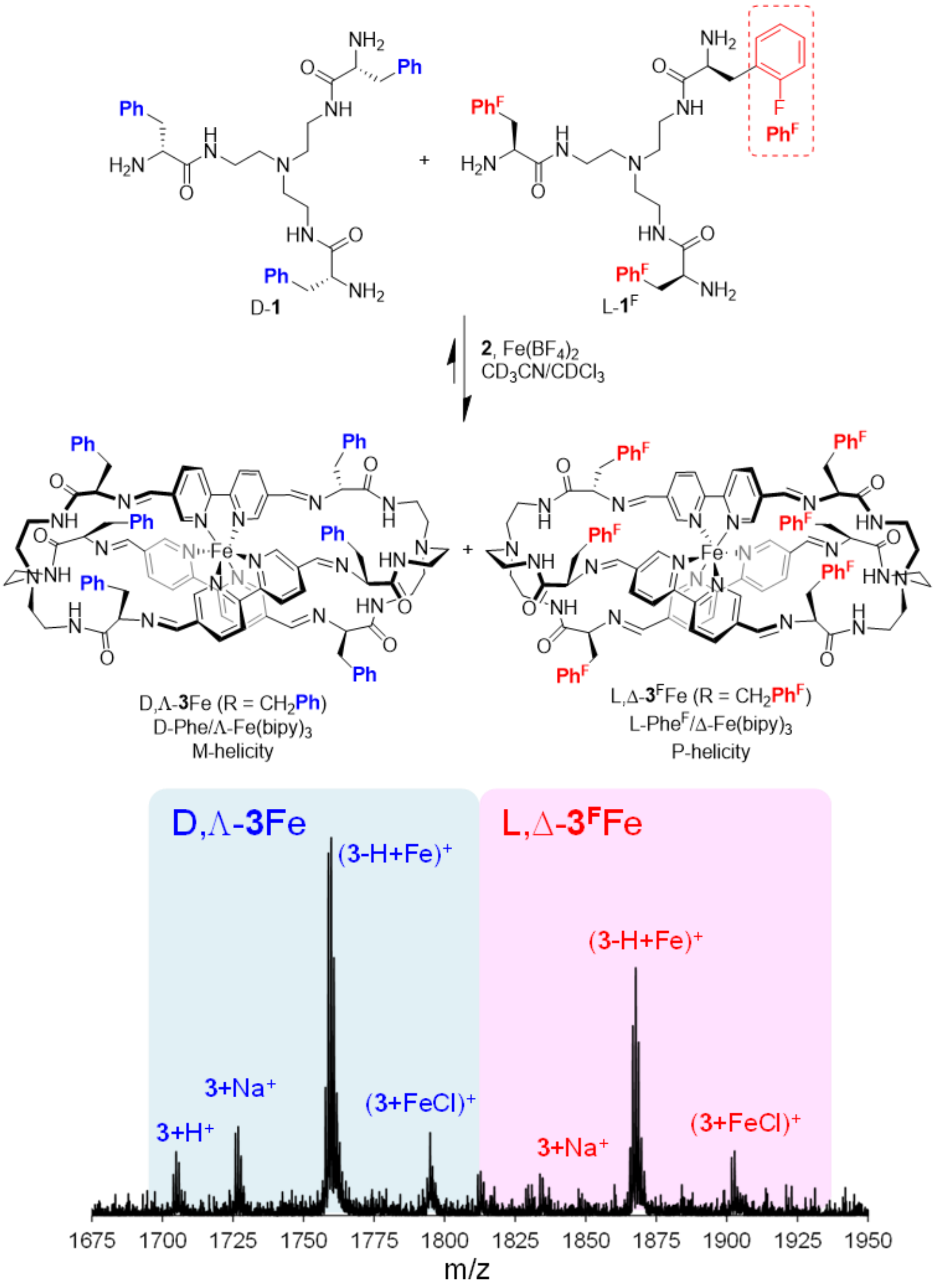

Figure S4.1. Stereospecific self-sorting experiment with D-**1** and *ortho*-F L-**1**^{F}. The regions where the mass peaks of the two homochiral cages appear are highlighted in blue (no F atoms) and red (six F atoms).

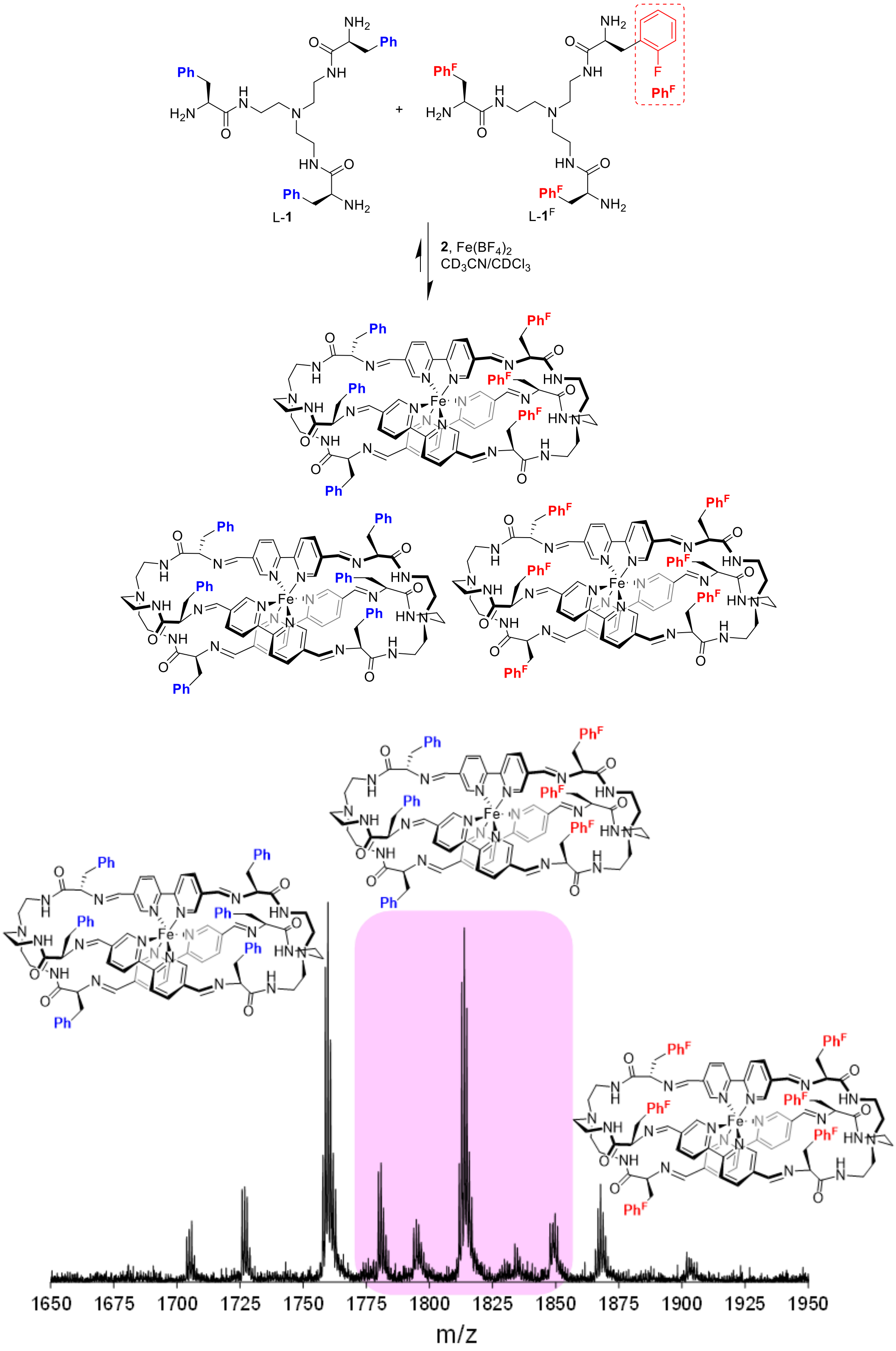

Figure S4.2. Control homochiral scrambling experiment with L-**1** and *ortho*-F L-**1**^{F}. The region where mass peaks corresponding to the mixed cage (three fluorine atoms) appear is highlighted.

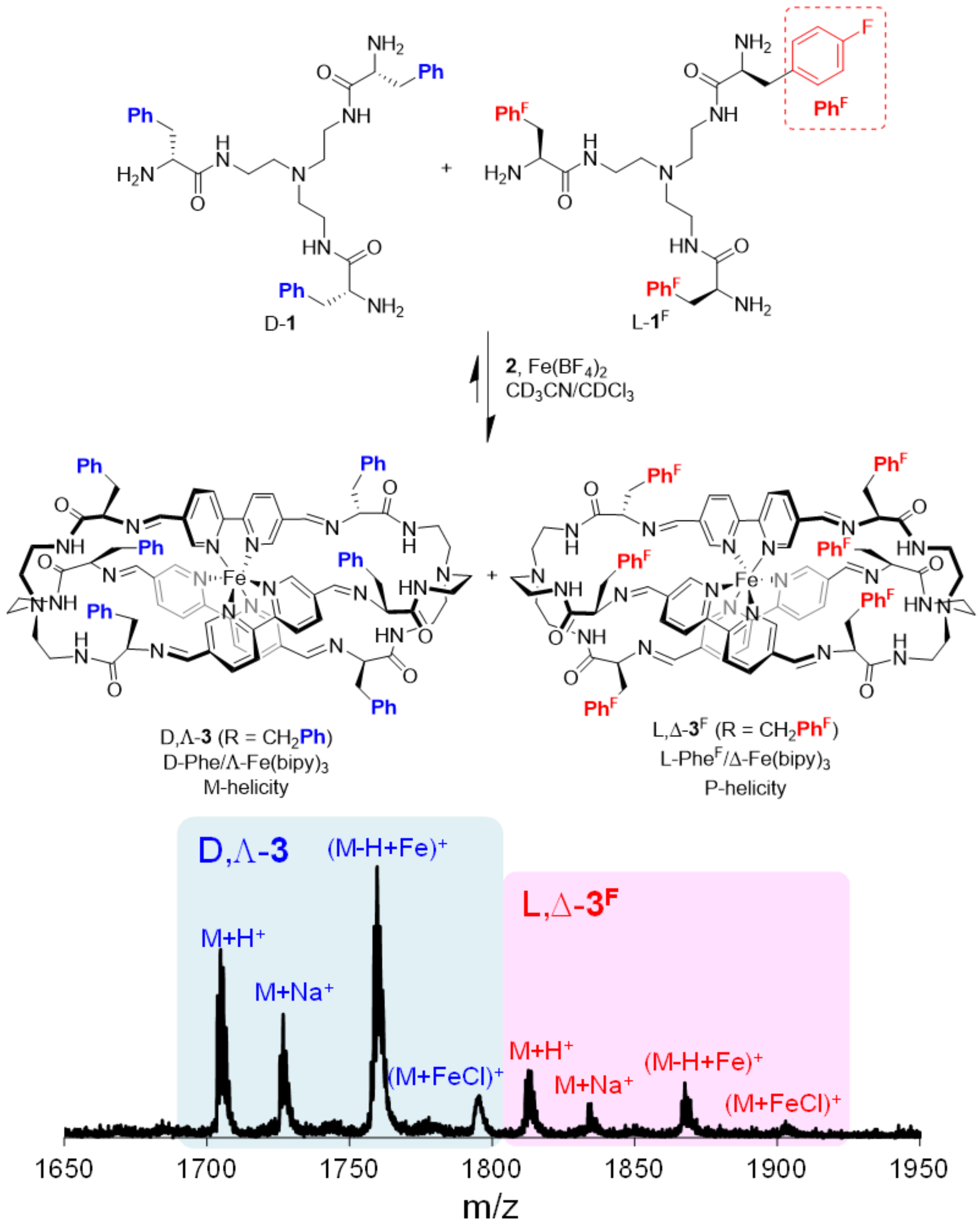

Figure S4.3. Stereospecific self-sorting experiment with D-**1** and para-F L-$\mathbf{1}^F$. The regions where the mass peaks of the two homochiral cages appear are highlighted in blue (no F atoms) and red (six F atoms).

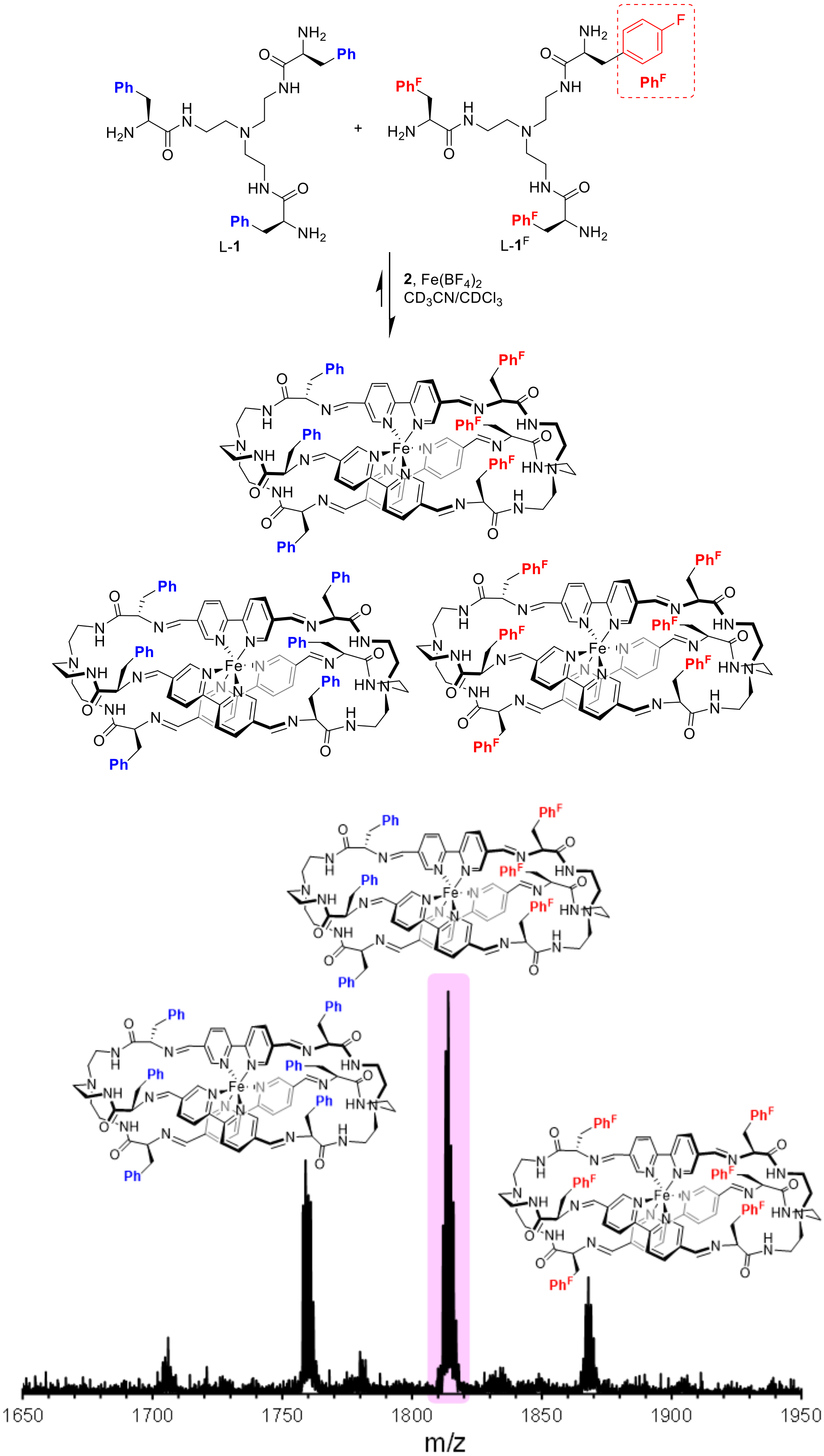

Figure S4.4. Control homochiral scrambling experiment with L-**1** and *para*-F L-**1**^{F}. The region where mass peaks corresponding to the mixed cage (three fluorine atoms) appear is highlighted.

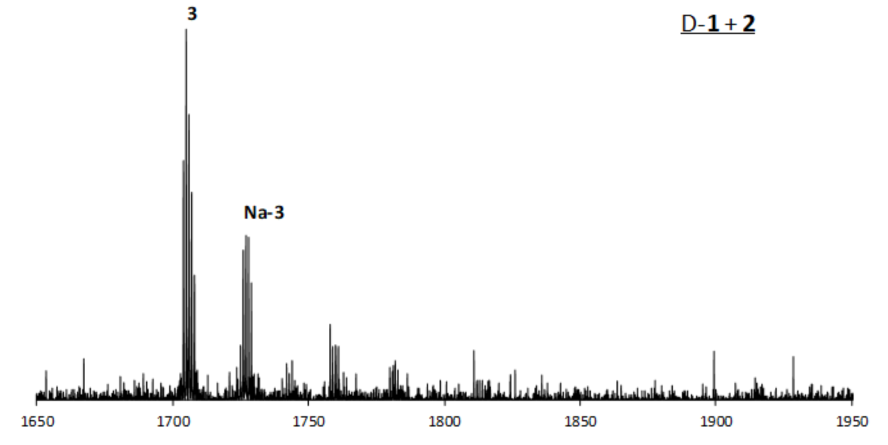

Figure S4.5. Control mass spectrum of D-**3** in absence of Fe(II)

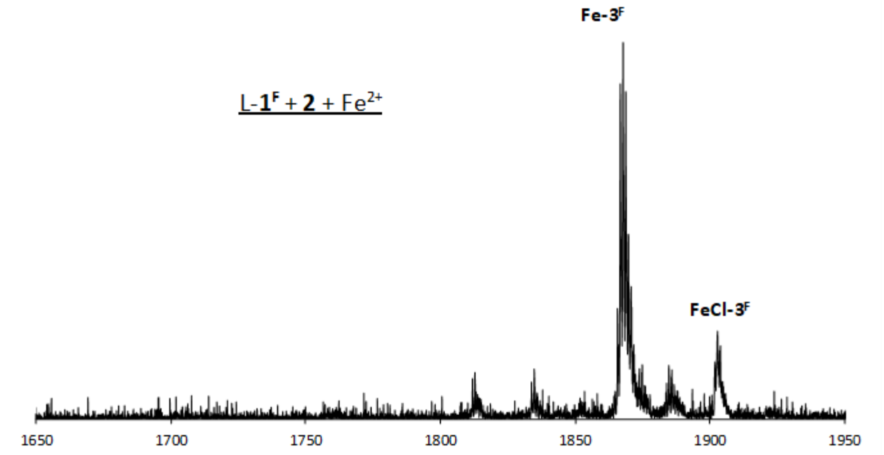

Figure S4.6. Control mass spectra of the pure fluorinated cage in the presence of iron(II), L-$\mathbf{3}^{F}$Fe.

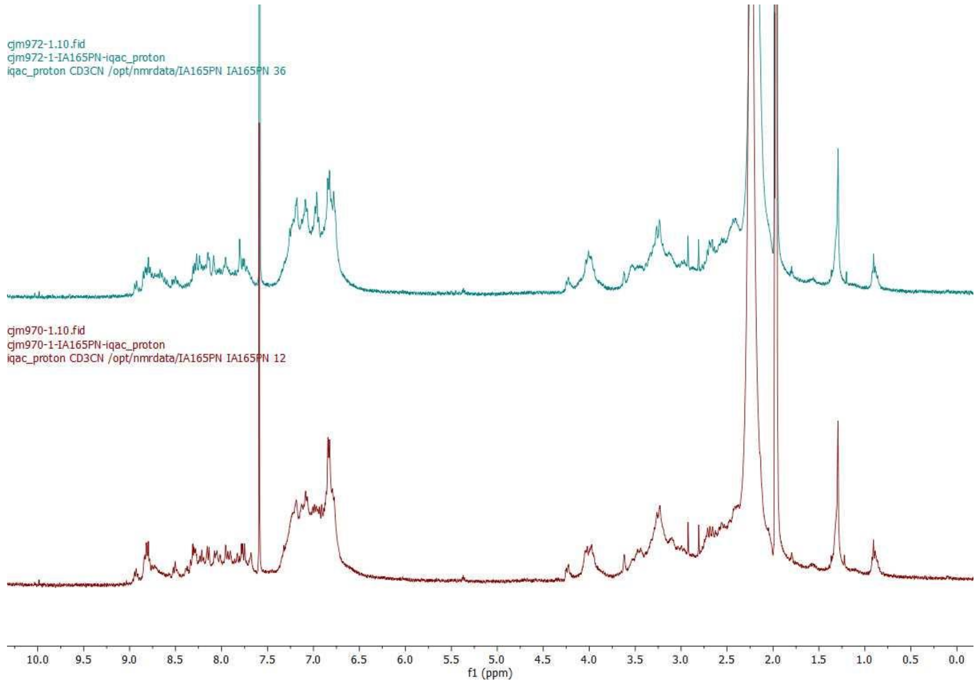

Figure S4.7. ^{1}H-NMR spectra of a D-**1** + L-**1**^{F} + **2** + Fe^{2+} mixture (top) and L-**1** + L-**1**^{F} + **2** + Fe^{2+} mixture (bottom) using the *p*-F-substituted ligand **1** under cage formation conditions (1.5 mM cage in $CD_3CN/CDCl_3$ 8/1)

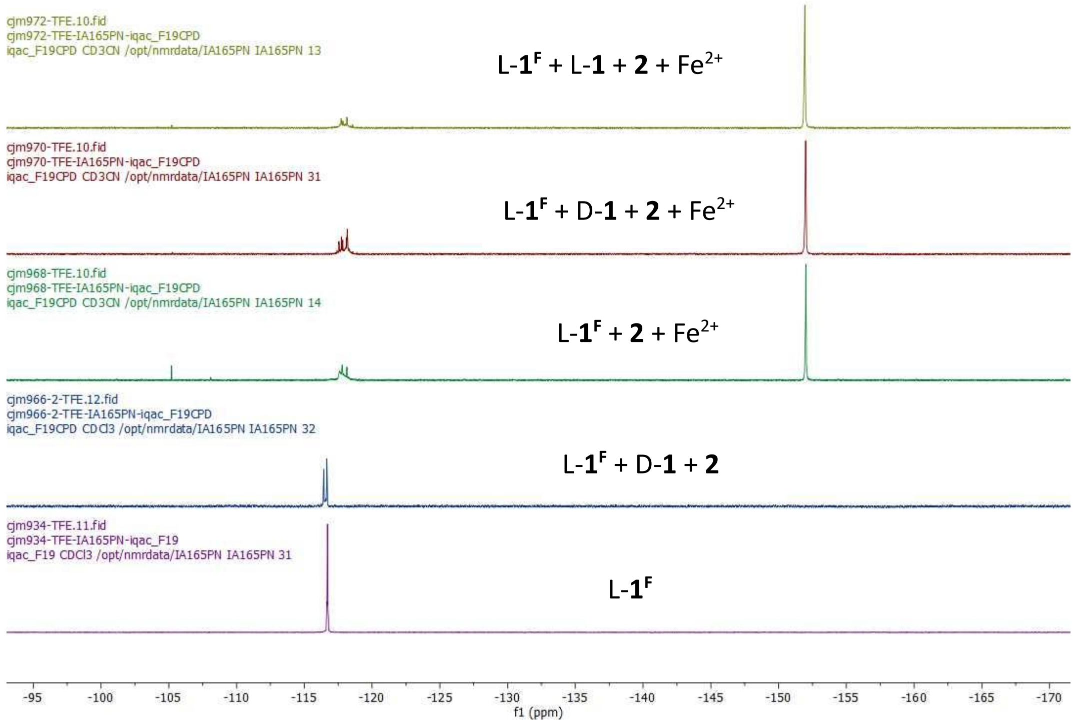

Figure S3.8. ^{19}F NMR spectra of diferent mixtures using trifluoroethanol as internal standard (not appearing in the spectral window shown) under cage formation conditions using the *p*-F-substituted ligand **1** (1.5 mM cage in $CD_3CN/CDCl_3$ 8/1).

5. XRD STRUCTURE AND CRYSTALLOGRAPHIC DATA OF **3**Fe

Crystal Data for $C_{105.5}H_{110.05}B_2Cl_2F_8FeN_{21.75}O_{6.4}$ (M =2085.47 g/mol): monoclinic, space group P2$_1$ (no. 4), a = 13.8986(3) Å, b = 16.7254(5) Å, c = 24.3794(7) Å, β = 101.556(3)°, V = 5552.3(3) Å^3, Z = 2, T = 100(2) K, μ(Mo Kα) = 0.259 mm^{-1}, $Dcalc$ = 1.247 g/cm^3, 43440 reflections measured (3.728° ≤ 2Θ ≤ 57.96°), 20673 unique (R_{int} = 0.0282, R_{sigma} = 0.0456) which were used in all calculations. The final R_1 was 0.0641 (I > 2σ(I)) and wR_2 was 0.1834 (all data).

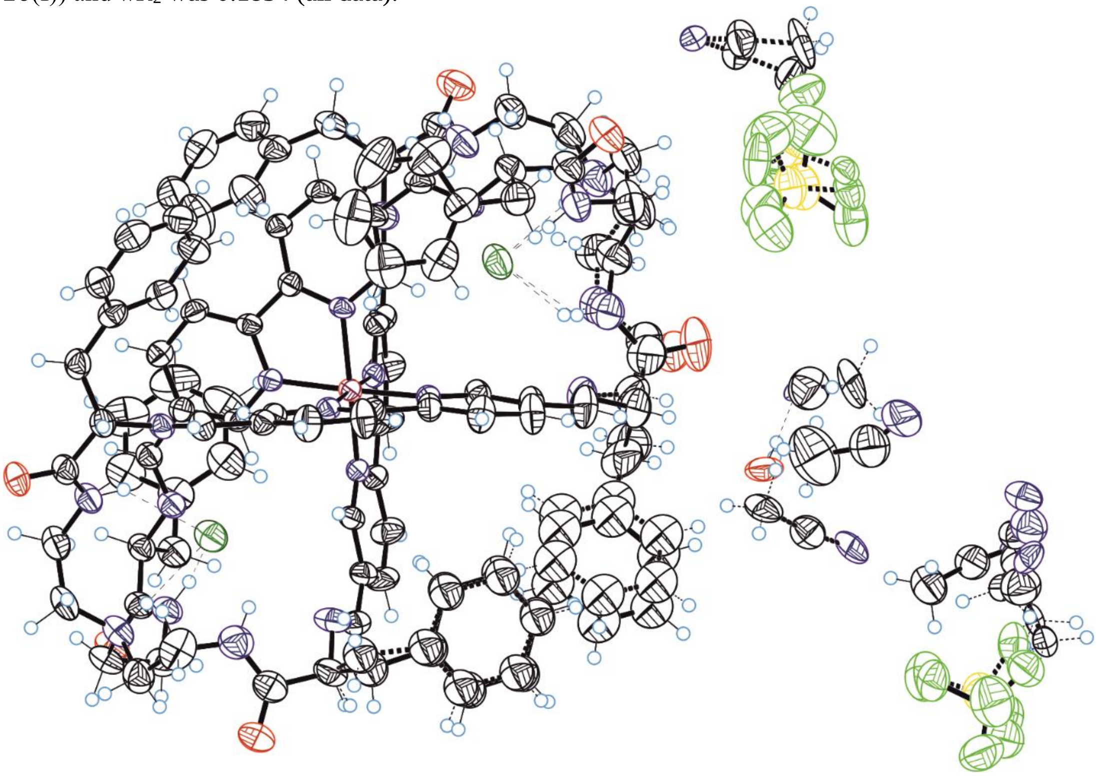

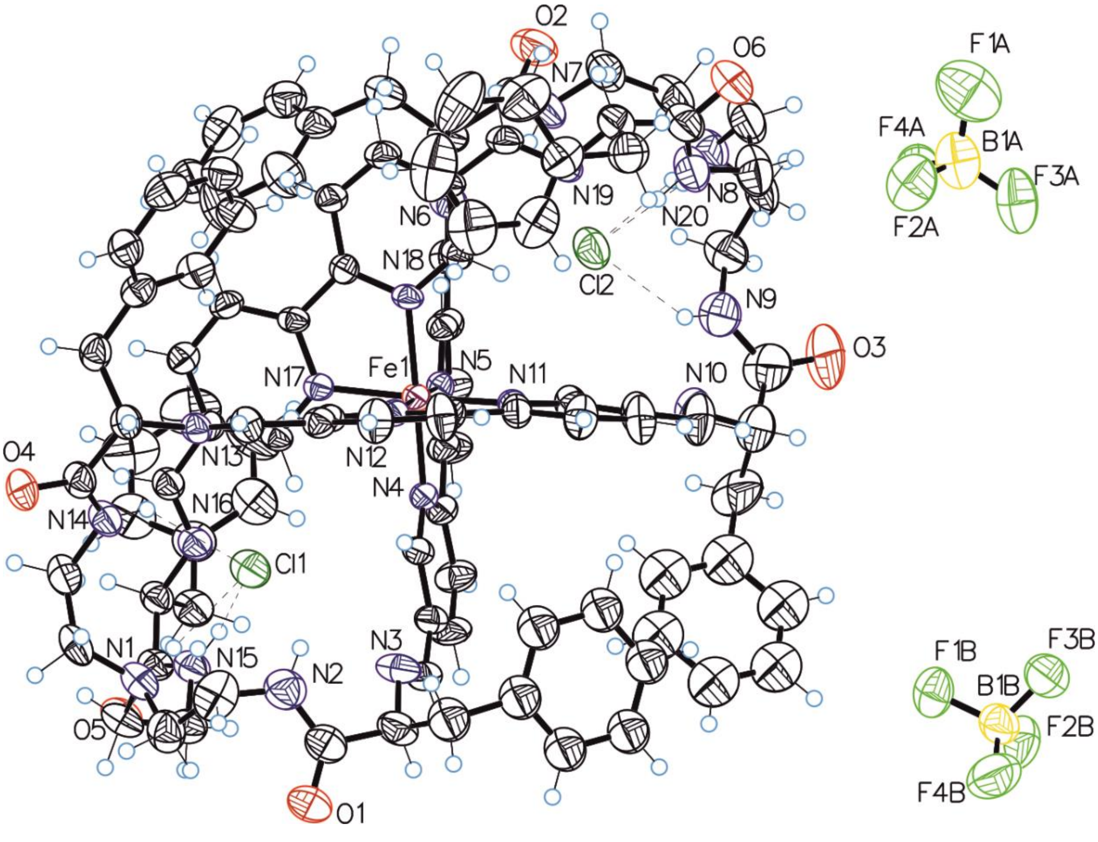

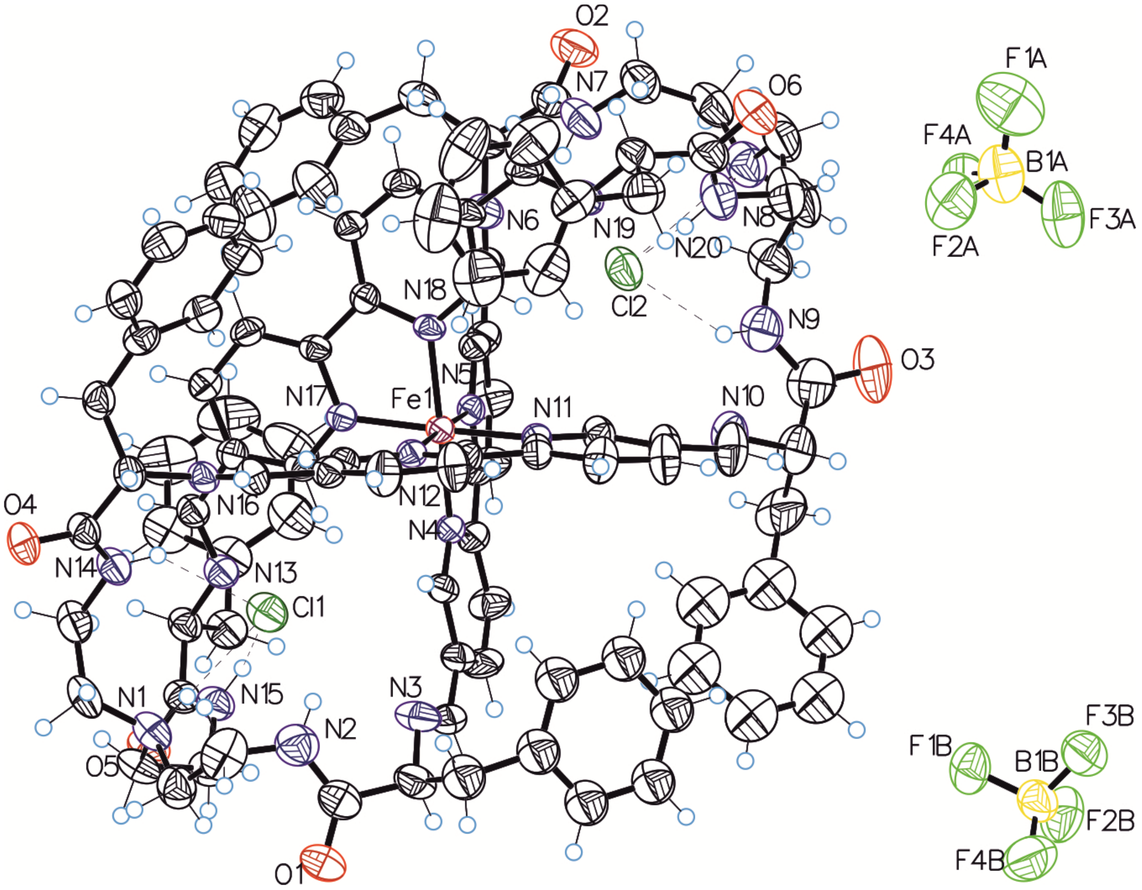

Figure S5.1. Ortep-plot figures (50 %) for the structure **3**Fe. The asymmetric unit contains one molecule of the cationic iron complex, two chlorine anions, two BF_4- anions, 1.75 molecules of acetonitrile and 0.4 molecules of water. The main molecule is partly disordered in two orientations (chain C35-45 ratio 77:23 and chain C5-C11 ratio 67:33). The acetonitrile molecules are disordered in 8 positions (ratio: 0.5:0.25:0.2:0.2:0.2:0.15:0.15:0.10). The water molecule is also located in the area of disordered solvent molecules with an occupancy of 0.4. The structure is of good quality (no A-alerts and two in the CIF commented be B-alerts) and publishable with a R1 value of 6.41 %.

Table 1 Crystal data and structure refinement for CJM881ALT_JBB72.

Identification code	CJM881ALT_JBB72
Empirical formula	$C_{105.5}H_{110.05}B_2Cl_2F_8FeN_{21.75}O_{6.4}$
Formula weight	2085.47
Temperature/K	100(2)
Crystal system	monoclinic
Space group	$P2_1$
a/Å	13.8986(3)
b/Å	16.7254(5)
c/Å	24.3794(7)
α/°	90
β/°	101.556(3)
γ/°	90
Volume/Å^3	5552.3(3)
Z	2
ρ_{calc}g/cm^3	1.247
μ/mm^{-1}	0.259
F(000)	2177.0
Crystal size/mm^3	0.2 × 0.16 × 0.05
Radiation	Mo Kα (λ = 0.71073)
2Θ range for data collection/°	3.728 to 57.96
Index ranges	$-16 \le h \le 18$, $-22 \le k \le 14$, $-31 \le l \le 32$
Reflections collected	43440
Independent reflections	20673 [R_{int} = 0.0282, R_{sigma} = 0.0456]
Data/restraints/parameters	20673/1148/1598
Goodness-of-fit on F^2	1.030
Final R indexes [I>=2σ (I)]	R_1 = 0.0641, wR_2 = 0.1682
Final R indexes [all data]	R_1 = 0.0854, wR_2 = 0.1834
Largest diff. peak/hole / e Å^{-3}	0.71/-0.51
Flack parameter	-0.008(6)

6. <u>THEORETICAL CALCULATIONS ON **3**Fe</u>

Table S6.1. B3LYP-D3 level of theory // LACVP basis set // acetonitrile PCM solvation mode

	Gas phase			Solution (PCM acetonitrile)		
Isomer	B3LYP-D3 LACVP**	ΔE (hartrees)	ΔE (kcal/mol)	B3LYP-D3 LACVP**	ΔE (hartrees)	ΔDE (kcal/mol)
L-TrenPhe// Δ-Fe(bipy)$_3$// D-TrenPhe	-5618.253411	0.010685	6.704869555	-5618.548883	0	0
D-TrenPhe// Δ-Fe(bipy)$_3$// L-TrenPhe	-5618.264096	0	0	-5618.505921	0.042962	26.95878389
D-TrenPhe// Δ-Fe(bipy)$_3$// D-TrenPhe	-5618.181624	0.082472	51.75142742	-5618.47909	0.069793	43.79531688

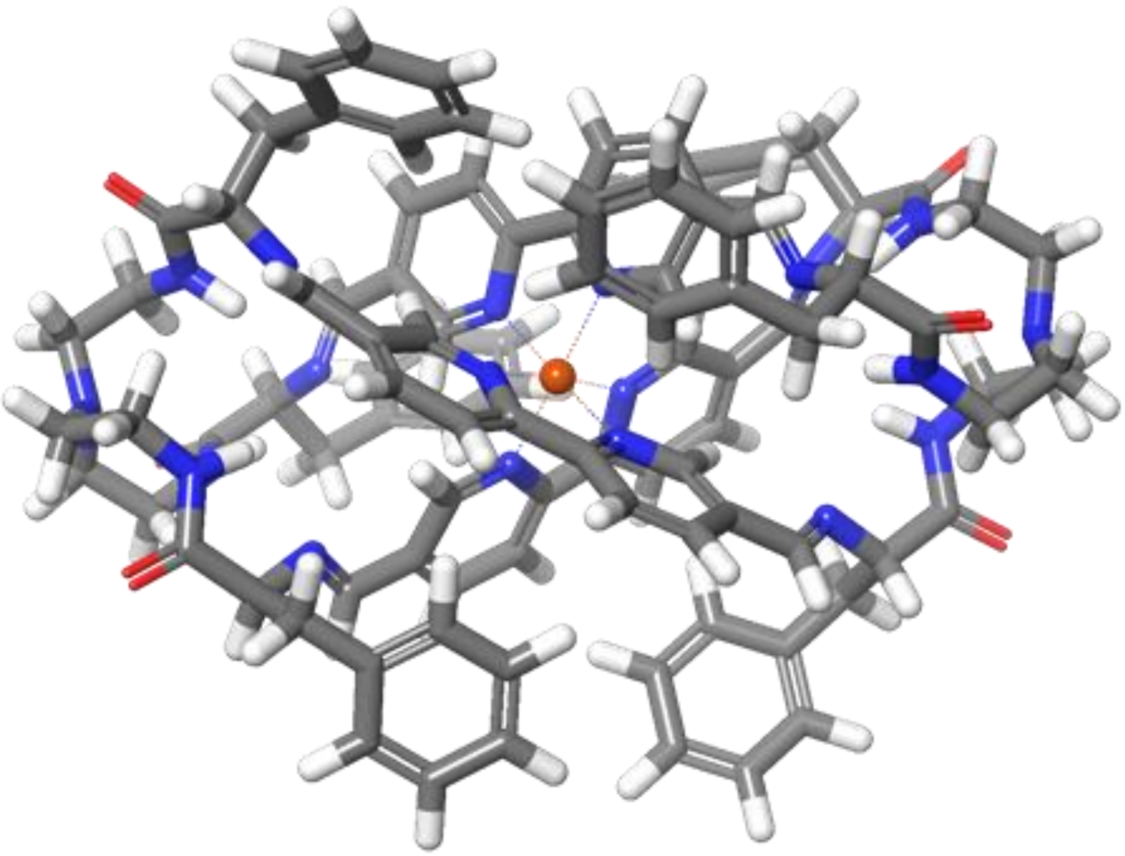

Figure S6.1. Structure of the computed geometries of the L,Δ,L experimental stereoisomer of **3**Fe at the B3LYP-D3 level of theory (LACVP basis set, acetonitrile PCM solvation mode).

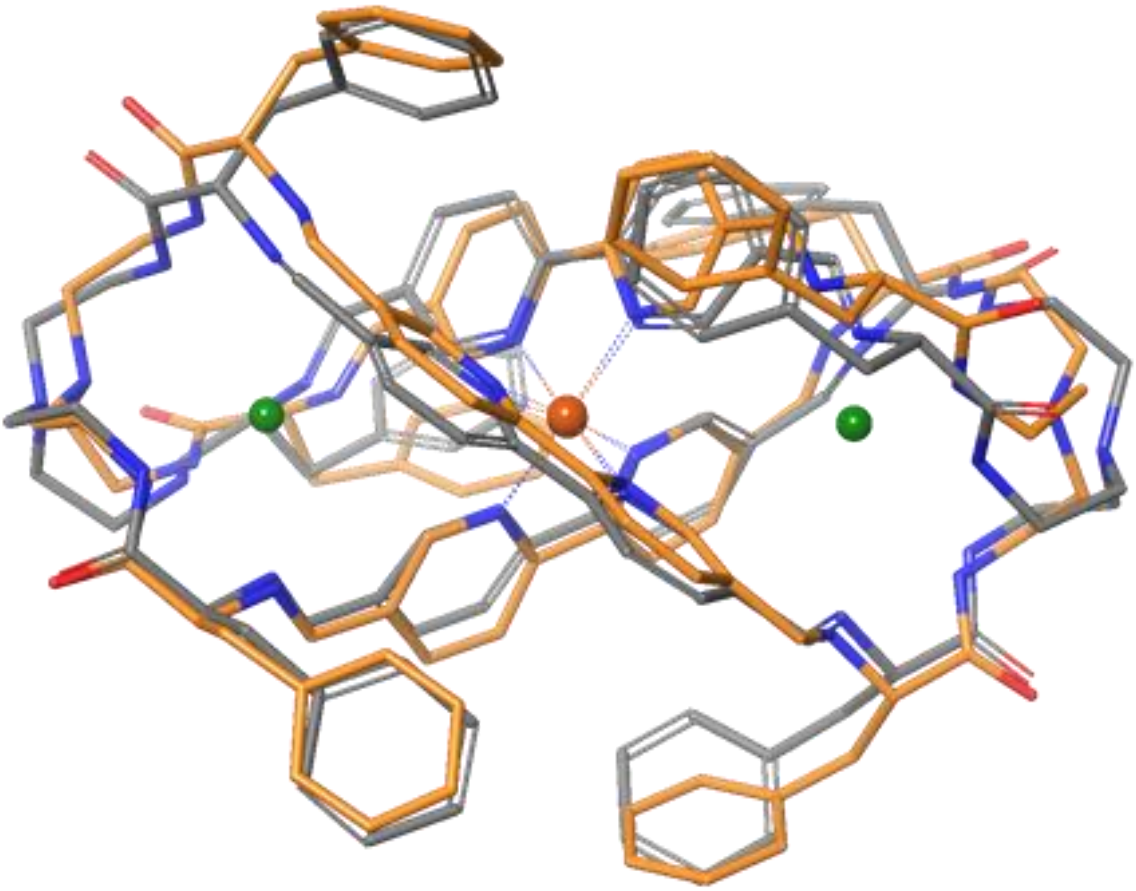

Figure S6.2. Overlayed DFT-optimized structure (grey C-atoms) with experimental X-ray diffraction structure (orange C-atoms). The experimental structure is more compressed along de C_3 symmetry axis due to an attractive interaction between the protonated tertiary amine and the chloride anions, as well as these with central iron (II). The two HCl molecules found in the crystal were obviated in the models, since they are not needed for stabilizing the cage in solution.

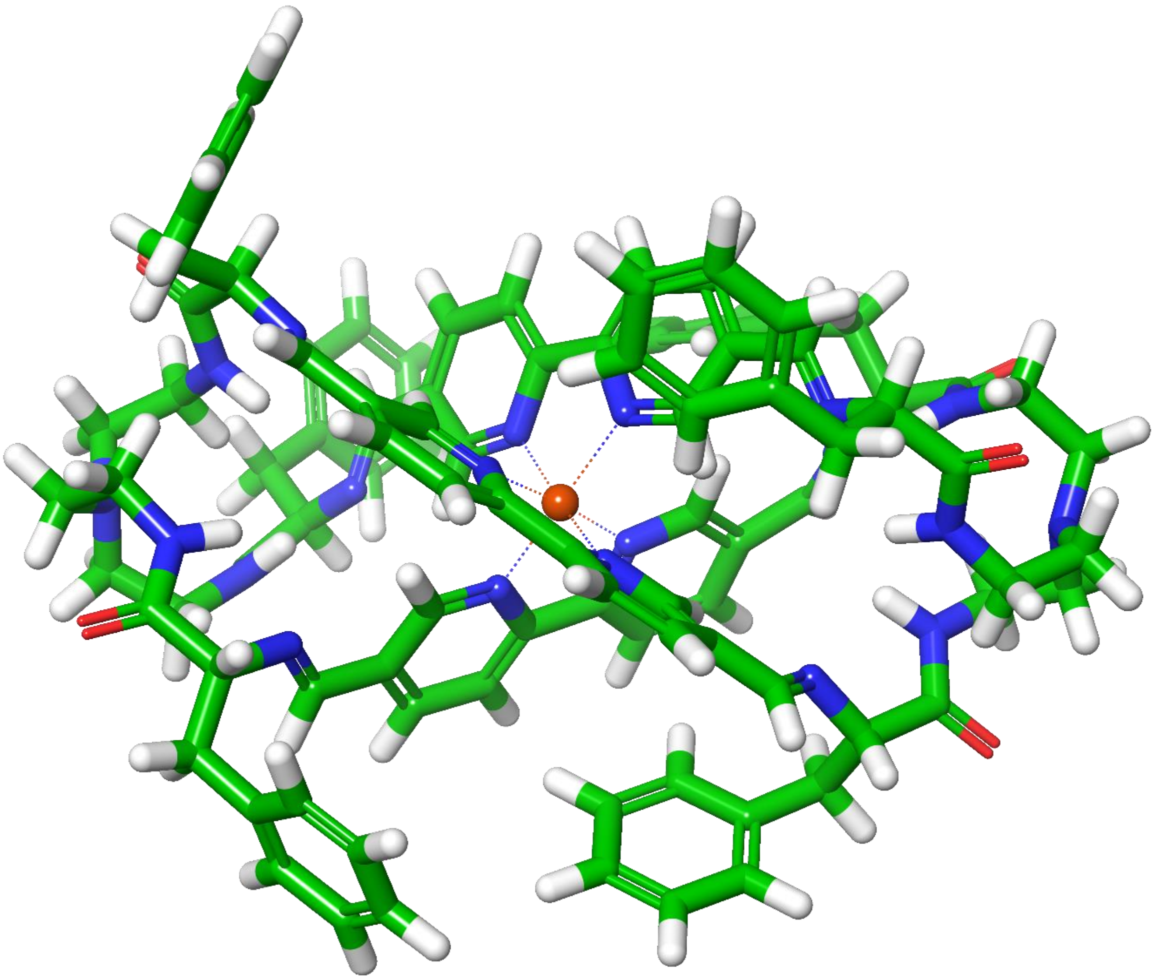

Figure S6.3. Structure of the computed geometries of the D,Δ,L stereoisomer of **3**Fe at the B3LYP-D3 level of theory (LACVP basis set, acetonitrile PCM solvation mode).

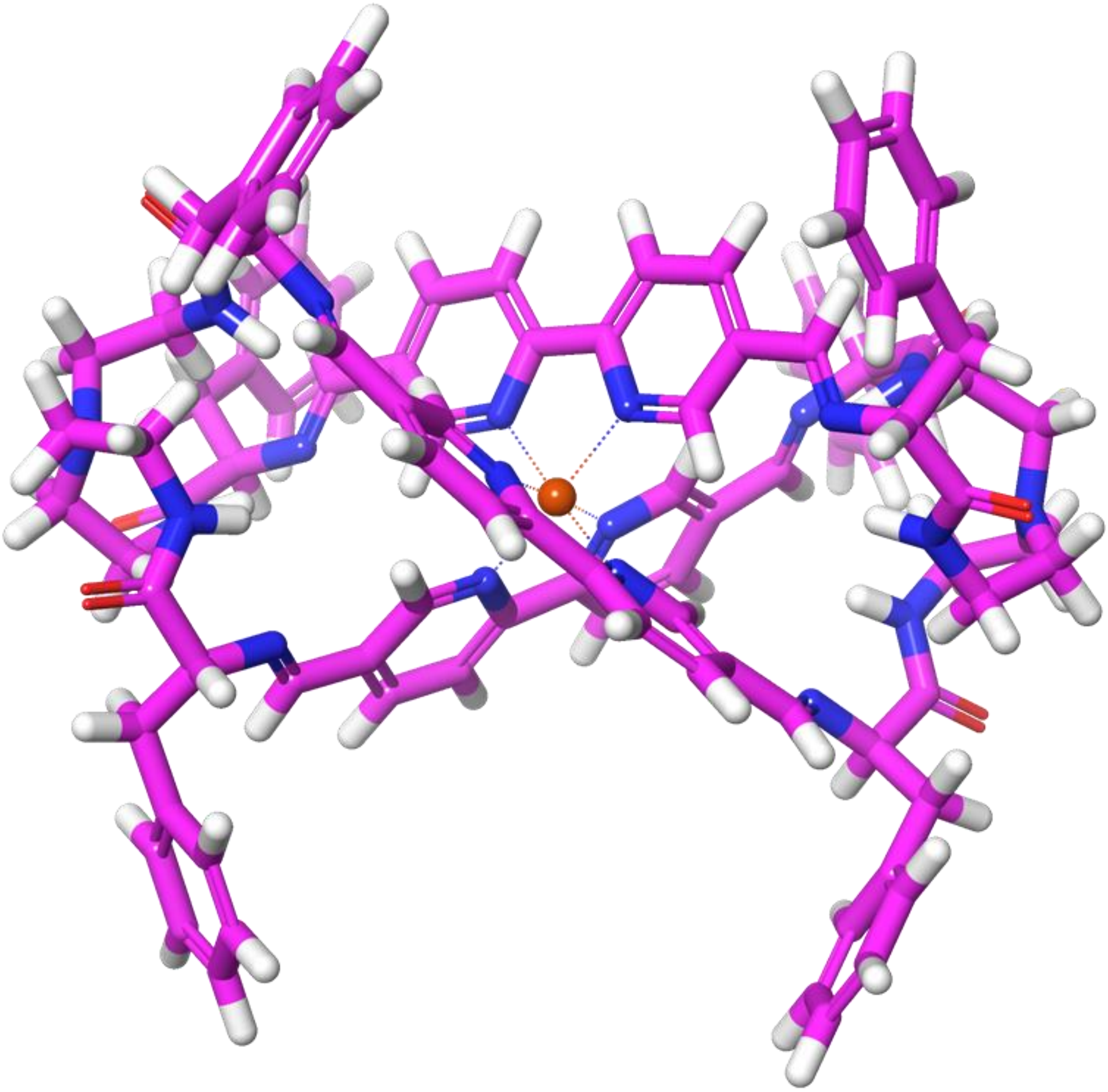

Figure S6.4. Structure of the computed geometries of the D,Δ,D stereoisomer of **3**Fe at the B3LYP-D3 level of theory (LACVP basis set, acetonitrile PCM solvation mode).

7. <u>SPECTROPHOTOMETRIC EXPERIMENTS WITH **5**Ru AT DIFFERENT pH</u>

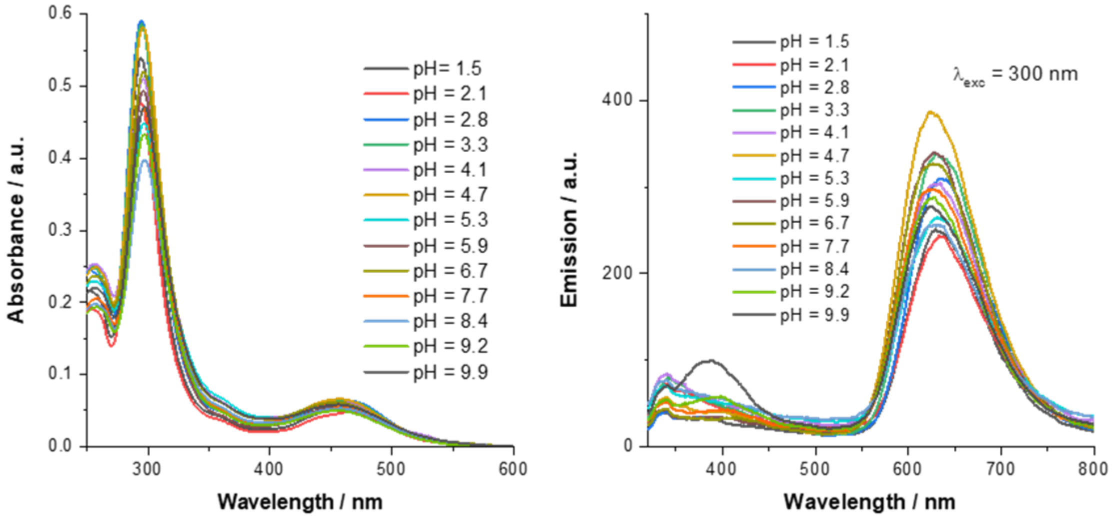

Figure S7.1. UV-Vis absorption(left) and fluorescence emission(right) spectra of **5**Ru at different pH values.

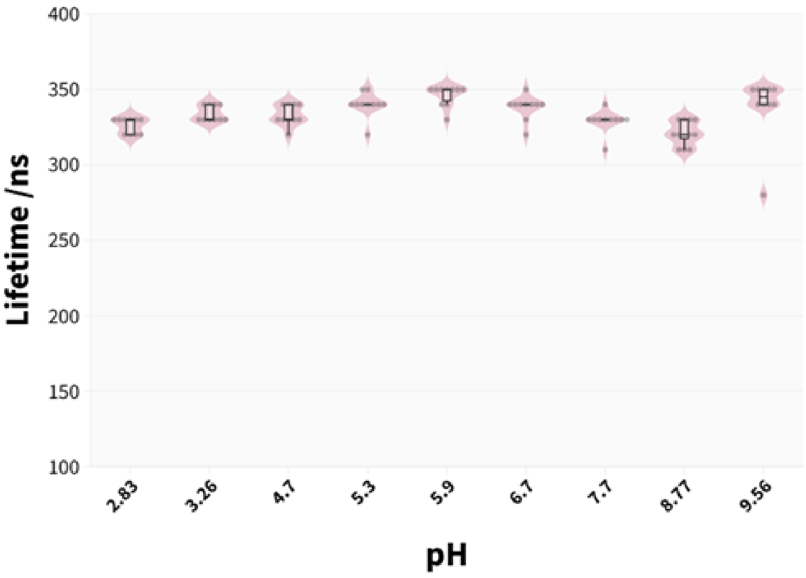

Figure S7.2. Lifetime of excited **5**Ru at different pH values.

8. REFERENCES

[1] G. M. Sheldrick, “{\it SHELXT} {--} Integrated space-group and crystal-structure determination” *Acta Crystallogr. Sect. A* **2015**, *71*, 3–8.

[2] O. V Dolomanov, L. J. Bourhis, R. J. Gildea, J. A. K. Howard, H. Puschmann, “OLEX2: a complete structure solution, refinement and analysis program” *J. Appl. Crystallogr.* **2009**, *42*, 339–341.

[3] G. M. Sheldrick, “Crystal structure refinement with {\it SHELXL}” *Acta Crystallogr. Sect. C* **2015**, *71*, 3–8.

[4] Y. Cao, T. Balduf, M. D. Beachy, M. C. Bennett, A. D. Bochevarov, A. Chien, P. A. Dub, K. G. Dyall, J. W. Furness, M. D. Halls, T. F. Hughes, L. D. Jacobson, H. S. Kwak, D. S. Levine, D. T. Mainz, K. B. Moore III, M. Svensson, P. E. Videla, M. A. Watson, R. A. Friesner, “Quantum chemical package Jaguar: A survey of recent developments and unique features” *J. Chem. Phys.* **2024**, *161*, 52502.

[5] Schrödinger, *Release 2024-2*, Schrödinger, LLC, New York, NY, **2024**.

[6] C. Lu, C. Wu, D. Ghoreishi, W. Chen, L. Wang, W. Damm, G. A. Ross, M. K. Dahlgren, E. Russell, C. D. Von Bargen, R. Abel, R. A. Friesner, E. D. Harder, “OPLS4: Improving force field accuracy on challenging regimes of chemical space” *J. Chem. Theory Comput.* **2021**, *17*, 4291–4300.

[7] S. T. Schneebeli, A. D. Bochevarov, R. A. Friesner, “Parameterization of a B3LYP Specific Correction for Noncovalent Interactions and Basis Set Superposition Error on a Gigantic Data Set of CCSD(T) Quality Noncovalent Interaction Energies” *J. Chem. Theory Comput.* **2011**, *7*, 658–668.
